# Radiation‐Induced Biological Effects: Molecular and Cellular Mechanism, and Applications to Radiation/Nuclear Emergency and Cancer Therapy

**DOI:** 10.1002/mco2.70478

**Published:** 2025-11-23

**Authors:** Zhihe Hu, Yan Chen, Shengyi Yang, Shihai Diao, Le Ma, Chunmeng Shi

**Affiliations:** ^1^ Institute of Rocket Force Medicine State Key Laboratory of Trauma and Chemical Poisoning Army Medical University Chongqing China; ^2^ Oncology and Hematology Department People's Liberation Army of China 96110 Hospital Yinchuan China

**Keywords:** biodosimetry, inflammation, radiation‐induced cell death, radiation injury, radiotherapy, senescence

## Abstract

Radiation has been wildly used in clinics for disease therapy over a century. However, the side effects associated with radiotherapy are often substantial and multifaceted, underscoring the need for effective management of radiation‐induced injuries. Additionally, with the growing reliance on nuclear technology, concerns about accidental exposures have intensified. Radiation‐induced biological effects represent a highly complex process that impacts multiple physiological activities within organisms. Ongoing research aims to elucidate the underlying molecular mechanisms and to develop reliable methods for radiation dose estimation—both for emergency scenarios and therapeutic applications. This review provides a systematic overview of the biological effects of ionizing radiation, covering DNA damage and repair pathways, cellular senescence, and diverse modes of cell death. It also offers an in‐depth analysis of recent advances in radiation biodosimetry and the application of radiation in cancer therapy, along with insights into future research directions. By integrating mechanistic knowledge with practical applications, this review aims to support the optimization of radiation‐based strategies, enhance public health preparedness, and inspire continued innovation in the field.

## Graphical abstract

Radiation‐induced biological effects are an extremely complex and extensive mechanism that involves multiple aspects of physiological activities in organisms. In the medical field, utilizing the damaging effects of radiation to treat tumors is a commonly employed therapeutic approach. Recent studies have also discovered that radiation exhibits potential therapeutic effects in neurological disorders such as Alzheimer's disease. However, with the continuous development of nuclear technology, the probability of nuclear accidents has increased, making rapid response to potentially large‐scale nuclear incidents a critical focus of current research. Numerous reports emphasize that radiation biodosimetry plays a pivotal role in nuclear emergency rescue operations. However, current radiation biodosimetry detection technologies face significant limitations. Traditional detection methods, such as dicentric chromosome analysis and micronucleus assay, generally encounter technical bottlenecks including limited throughput and time‐consuming detection processes. Therefore, there is an urgent need to develop novel radiation biodosimetry systems with high‐throughput and rapid response characteristics. This review systematically and comprehensively summarizes the mechanisms of ionizing radiation's biological effects, provides an in‐depth and detailed analysis of the latest research progress in radiation dose estimation and medical applications, and offers insights into future development trends and prospects. It aims to provide solid scientific theoretical support and practical guidance for optimizing radiation application strategies and safeguarding public health security, while promoting scientific research and technological innovation in related fields to new heights.

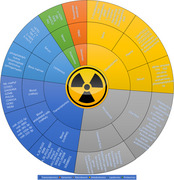

## 1 Introduction

Since Henri Becquerel's discovery of radioactive substances in 1896, ionizing radiation (IR) has found extensive applications in agriculture, as food preservation, storage, and breeding practices; industry, such as non‐destructive testing and power generation; and medicine, encompassing diagnostic imaging and radiation therapy [1]. Despite these evident societal benefits, health hazards arising from IR exposure in clinical and public health practices remain a pressing challenge [1]. Over the past century, radioactive waste from nuclear energy and nuclear weapons programs has accumulated continuously. Radioactive fallout containing isotopes such as ^90^Sr, ^14^C, and ^137^Cs from weapons testing and nuclear accidents, such as Chernobyl, Mayapuri, and Fukushima, along with the potential threats of nuclear warfare or radiological terrorism, underscore the risks of mass casualties, including acute radiation syndrome (ARS) [2]. Beyond nuclear accidents, workers exposed occupationally and patients undergoing radiation therapy or CT imaging may be exposed to various forms of IR. These circumstances keep radiation risks under constant scrutiny by both the scientific community and the public.

Ionizing radiation encompasses high‐energy photons such as γ‐rays and X‐rays, as well as subatomic particles including α particles, β particles, and neutrons. Different types of radiation exhibit variations in penetrating power and linear energy transfer: α particles possess strong ionizing properties but weak penetration, β particles penetrate deeper but have lower ionizing density, and γ rays can easily penetrate the human body [3]. On Earth, α particles and β particles can be shielded by paper and aluminum, respectively; however, in space, the higher particle energies make effective shielding more challenging [4]. Absorbed dose is measured in Gray (Gy), but biological effects are better assessed using effective dose in Sievert (Sv), which accounts for radiation type and tissue weighting factors [5]. Short‐term exposure to approximately 1 Sv can induce acute radiation sickness, which manifests as hair loss, nausea, and erythema. Exposure to 2–10 Sv may result in significant patient mortality, while doses exceeding 10 Sv are typically fatal without hematopoietic stem cell transplantation [6]. These outcomes fundamentally arise from the biological mechanisms of radiation: ionization tracks from radiation can directly damage DNA, lipids, and proteins [7], while hydroxyl radicals generated by radiation‐induced water cleavage mediate indirect biomolecular damage, accounting for approximately two‐thirds of radiation‐induced DNA damage [8]. Unrepaired or incorrectly repaired damage drives diverse cellular fates depending on dose and environment. These include apoptosis, ferroptosis, pyroptosis, necrosis, and other non‐apoptotic deaths, as well as nonlethal outcomes such as cellular senescence and mitotic catastrophe [9].

The biological effects of IR have laid the foundation for disease control and rapid radiation dose assessment. Taking advantage of the difference in radiosensitivity between tumor and normal cells and combined with the modulation of signaling pathways related to radiation response, it is expected that tumor radiosensitivity can be enhanced while damage to healthy tissues is reduced. Dosimetric methods utilizing the biological effects of radiation for radiation dose assessment in nuclear accidents, such as the dicentric chromosome assay (DCA), micronucleus test (MN), fluorescence in situ hybridization (FISH) translocation test, premature chromosome condensation (PCC), and lymphocyte proliferation assay, have been applied in practice [10]. However, existing methods for rapid screening in the presence of a large number of casualties still have limitations, especially due to the time required for blood sample culture and the labor involved in result interpretation [11]. Constrained by these factors, a series of newly developed markers of radiation exposure, including γ‐H2AX foci assay, gene methylation, non‐coding RNA (ncRNA) expression, and transcriptome assays, have shown potential for radiation dose assessment at the preclinical level in recent years [12]. Particularly, based on high‐throughput and multi‐omics tools, these methods promise to assess radiation dose in a large number of samples within a shorter period.

Given these advances, this review synthesizes recent progress in the mechanisms of biological effects of IR, evaluates developments in radiation biodosimetry for nuclear‐emergency response, and surveys medical applications in oncologic settings. This review adopts a logical framework structured around three key areas: mechanisms of biological effects of IR, radiation dosimeters in nuclear accidents, and therapeutic applications exploiting the biological effects of IR. Its aim is to clarify conceptual frameworks, highlight opportunities for translation, and provide decision‐making support to optimize strategies in radiation protection and clinical practice.

## 2 Mechanisms of Radiation‐Induced Biological Effects

This section focuses on the multiscale biological effects induced by IR, extending from primary DNA damage and DNA damage response (DDR) to cellular fate outcomes, ultimately revealing the remodeling mechanisms at the tissue and systemic levels. First, we comprehensively describe the mechanisms of DNA damage induced by IR. Further elaborating on cellular DDR pathways, linking these molecular processes to cellular fates including apoptosis, necrotic cell death, pyroptosis, ferroptosis, and cellular senescence. In the end, for biomarker discovery and intervention strategies, we position these events within the inflammatory, immune, and microenvironmental circuits that regulate long‐term consequences such as repair and fibrosis.

### 2.1 Molecular Mechanisms: DNA Damage and Repair

**FIGURE 1:**
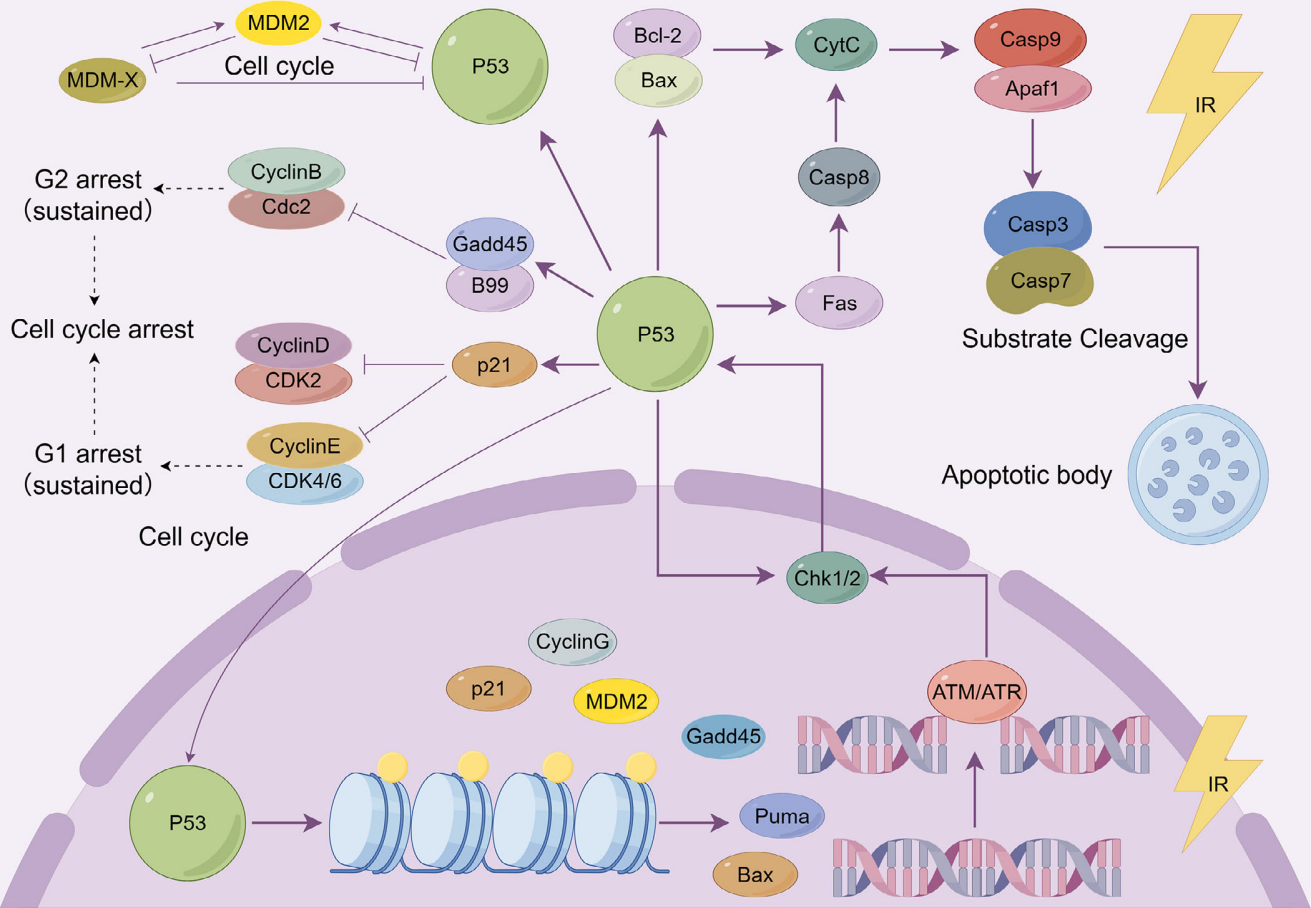
Molecular and cellular mechanisms of radiation‐induced damage. Schematic diagram of radiation‐induced apoptosis, cell cycle arrest, and DNA damage repair through the activation of the P53 pathway.

It has been reported that every 1 Gy of γ‐radiation produces about 1000 DNA single‐strand breaks (SSB), 40 DNA double‐strand breaks (DSBs), and 1300 DNA base lesions. These DNA damages are mainly produced through indirect mechanisms and the probability of damage is related to oxygen tension [13]. The DDR is a sophisticated signaling network that is used in cells to detect and repair a range of different DNA lesions. The core architecture of this network encompasses three crucial hierarchical levels: the identification of damage sites, the execution of repair commands, and the determination of cell fate. The schematic diagram of radiation‐induced cell cycle arrest and DNA damage repair is shown in Figure 1.

#### 2.1.1 Base‐Excision Repair

Oxidized DNA base damage, SSBs, and abasic (AP) sites are repaired primarily by the BER pathway. BER is initiated by a family of damage‐specific DNA glycosylases, including proteins such as OGG1, NTH1, and NEIL1‐3 [14]. These enzymes excise the damaged bases, and the DNA backbone is cleaved by APE‐1 after excision, and the residual 5′‐deoxyribose phosphate (dRP) is removed. SSBs produced as BER intermediates or directly are recognized by PARP‐1, which protects DNA single strands from breaks. Finally, Polβ and XRCC1‐Lig IIIα attach the SSB nick [15].

#### 2.1.2 Non‐Homologous End Joining

DSB repair begins with chromatin loosening and recruitment of repair factors. Deamidation modifications linking asparagine 76 and 77 (H1N76/77) sites in histone H1 promote acetylation of histone H1K75 through a cascade reaction, leading to chromatin loosening and promoting recruitment of damage repair factors such as ATM and ATR enzymes [16] and phosphorylation of the histone variant H2AX [17]. DSBs are usually repaired by the non‐homologous end joining (NHEJ) [18] or homologous recombination (HR) pathways. NHEJ can be categorized into two approaches, classical NHEJ (cNHEJ) and alternative NHEJ (aNHEJ). cNHEJ begins with the binding of the Ku70/Ku80 heterodimer, which recruits DNAPKcs and Artemis [19], followed by nucleotide addition by DNA polymerase μ, DNA polymerase λ, and DSB ligation by a complex composed of XRCC4, Lig IV, and XLF [20]. In aNHEJ, the MRE11‐RAD50‐NBS1 (MRN) complex synergistically resects DNA ends with CtIP, generating 3′‐overhangs and microhomologous regions [21]. PARP‐1 is recruited with DNA polymerase θ, which ligates the DNA ends by XRCC1 Lig IIIα or Lig I. The DNA ends are ligated by XRCC4, Lig IV, and XLF.

#### 2.1.3 Homologous Recombination

In S and G2 phases, DSBs can also be repaired by HR [22], which uses sister chromatid to ensure the accuracy [23]. After excision of the DNA ends by the MRN complex, the 3′ single‐stranded DNA tails are protected by RPA, which is subsequently replaced by BRCA2/RAD51. BRCA2/RAD51 initiates homology search and strand nucleofilament invasion using the sister chromatid. DNA synthesis and ligation generate a Holliday junction, which is then processed by resolvases.

### 2.2 Cellular Responses: Senescence and Various Cell Death Pathways

Depending on the severity of cellular damage, there are differences in cell fates after radiation. High doses of radiation may cause cell death, including apoptosis, necroptosis, pyroptosis, and ferroptosis [1]. When the dose has not yet reached a lethal dose and DNA damage persists, the cell faces permanently cell cycle arrest, which is cellular senescence [24]. When the dose is light and the cell has fully completed repair of the DNA damage, the pre‐irradiation cellular state can be restored. Each of the multiple fates of cells after radiation will be reviewed below.

#### 2.2.1 Senescence

Senescent cells are a class of cell populations that enter a permanent proliferative arrest, characterized by persistent secretion of senescence‐associated secreted phenotypes (SASPs), metabolic reprogramming, and activation of anti‐apoptotic pathways [25]. Cellular senescence can be triggered by oxidative stress, oncogene‐induced stress, mitochondrial dysfunction, and DNA damage therapies, with IR being one of the most important causes of induced cellular senescence [26]. After IR damages DNA, activated p53 gene upregulates the expression of p21 gene, which in turn inhibits the expression of CDK–cyclin complex, inhibition of CDK 1–cyclin A complex blocks cells in G2 or M phase, and inhibition of CDK 2–cyclin E/A complex and CDK 4–cyclin D complex promotes the dephosphorylation of pRB and blocks the cells in G1 or S phase. In addition, IR‐induced elevation of intracellular reactive oxygen species (ROS) and protein kinase C (PKC) also increases p16 expression, inhibits CDK‐cyclin complexes, and promotes pRB dephosphorylation, leading to cellular senescence [27].

Senescent cells secrete a large number of SASPs, including chemokines, pro‐inflammatory cytokines, growth factors, and matrix metalloproteinases (MMPs), which lead to a wide range of potent biological effects in senescent cells. SASPs are highly heterogeneous, depending on the cell type and the mode of senescence induction. SASP regulation occurs at multiple levels, including transcription, translation, mRNA stability, and secretion, which results in differences in SASP at different stages of senescence [28]. Key transcriptional regulators include transcriptional levels of nuclear factor κB (NF‐κB) and CCAAT/enhancer‐binding protein β (C/EBP‐β) transcription factors [29]. mTOR increases the activity of C/EBPβ and NF‐КB and promotes the translation of a variety of inflammatory factors, which positively and feedback amplifies SASP [30], leading to post‐transcriptional regulation of SASP.

Numerous studies have observed the presence of senescent cells in oncological radiotherapy and radiation‐associated diseases. Wang et al. previously reported that hematopoietic stem cells from whole‐body irradiated mice underwent senescence as evidenced by the upregulation of p16 and SA‐β‐gal in comparison to irradiated hematopoietic progenitor cells that showed no changes in these biomarkers [31]. It has also been reported that whole‐body radiation can also cause vascular endothelial cells to undergo senescence, as evidenced by upregulation of the senescence biomarkers SA‐β‐gal, p16, and p21 [32]. Localized lung radiation can also induce senescence in alveolar epithelial cells, alveolar stem cells, and mesenchymal stem cells [33]. In brain exposure, researchers have observed upregulation of the expression of senescence‐related markers in the hippocampus and cortex [34] (p21), while neural stem cells can also undergo senescence as evidenced by an increase in the expression of p16, γ‐H2AX, ROS, and the SASP factor Il‐6 [35]. Large numbers of senescent cells have also been observed in radiation‐induced skin injury [36]. Large numbers of senescent cells can be induced after radiation were observed using organoids, primary cells [37]. In tumors, it has been shown that malignant gliomas undergo significant senescence of astrocytes after radiotherapy, as evidenced by increased expression of p16 and p21, as well as a massive secretion of SASP factors such as HGF and IL6 [38]. Targeted clearance of radiation‐induced senescence using Bcl‐2 inhibitors attenuated glioblastoma recurrence [39]. Injection of IR‐induced senescent human glioblastoma multiforme cells into nude mice resulted in more rapid tumor growth compared to unirradiated non‐senescent cells [40]. This suggests that these cells have a “double‐edged sword” role in physiological and pathological processes. It is currently believed that short‐term presence of senescent cells can promote tissue repair and attenuate collagen fiber production through secretion of SASP, and also recruit immune cells to accelerate tumor cell clearance, while long‐term accumulation of senescent cells exacerbates chronic inflammation, promotes tissue fibrosis, inhibits tumor local immune cells, and inhibits tumor growth, fibrosis, and tumor local immune cell activity [41].

These evidence suggest that radiation‐induced senescence in both tumors and normal tissues contributes to tumor recurrence, metastasis, and resistance to therapy, and that senescent cells in normal tissues and organs are the source of many of the delayed damaging effects. Exploiting the cellular and molecular pathways of cellular senescence holds the promise of increasing the sensitivity of tumor cells to radiotherapy and attenuating radiation‐induced adverse effects.

#### 2.2.2 Apoptosis

Apoptosis is a silent, non‐immunogenic form of programmed cell death and is the major pathway for cell renewal in organismal normality, which was originally proposed by Willie et al. in 1972 [42]. Morphologically, apoptotic cells exhibit cellular crumpling, nuclear consolidation, and cell membrane blistering [42]. It is mediated by two main types of pathways: the exogenous death receptor pathway or the endogenous mitochondrial pathway [43]. The exogenous pathway is initiated by ligand activation of members of the tumor necrosis factor receptor family such as Fas and TNF‐R1, which activate the caspase cascade reaction [43]. The endogenous pathway is triggered by intracellular stress signals that activate BAX/BAK to form a pores in the external mitochondrial membrane, resulting in cytochrome C release and activation of caspase‐9 [1]. These two pathways ultimately lead to the co‐activation of caspase‐3 and caspase‐7, which initiates apoptosis [1]. Ionizing radiation ultimately leads to caspase‐3/‐7 activation and apoptosis by inducing ceramide production, p53 activation, and death receptor signaling.

Radiation‐induced ceramide synthesis is one of the well‐studied key mechanisms of apoptosis. Sphingomyelinase hydrolyzes the phosphodiester bonds of membrane sphingolipids and produces ceramide [44]. Radiation could trigger ceramide production within minutes after exposure [44]. In vitro studies have shown that exogenous addition of ceramide analogs is sufficient to induce apoptosis in endothelial cells, and exposure of cells with only their nuclei removed similarly promotes sphingomyelin hydrolysis and production of ceramides [45], suggesting that radiation‐induced apoptosis due to ceramides is independent of DNA damage. In vivo studies have confirmed that prophylactic or therapeutic use of anti‐ceramide antibodies in mouse models of radioactive intestinal injury are effective in reducing radiation injuries and improving survival [46]. In a mouse model of acute radiation disease with 15 Gy whole‐body irradiation, anti‐ceramide treatment was effective in improving survival [47]. Systemically irradiated sphingomyelinase knockout mice showed a significant reduction in the number of apoptotic cells in several organs compared to wild‐type controls [48]. These results suggest that radiation induces ceramide production in a DNA damage‐independent manner and induces apoptosis following radiation.

p53 drives the endogenous apoptotic pathway mainly through the activation of pro‐apoptotic factors such as BAX and BAK, which ultimately mediate mitochondrial cytochrome C release and caspase‐3/7 activation [49]. The pro‐apoptotic proteins BAX and BAK are key performers in mediating mitochondrial outer membrane permeabilization (MOMP) and cytochrome C release. Radiation induces BAX/BAK aggregation in mitochondria, cytochrome C release, and caspase‐3 activation [50]. In vivo studies have shown that intestinal epithelial‐specific deletion of BAX and BAK is significantly resistant to radiation‐induced gastrointestinal syndrome [51]. Cytochrome C knockout or pharmacologically inhibited embryonic fibroblasts were resistant to radiation‐induced apoptosis and caspase‐3/7 activation [52]. In in vivo experiments, administering cytochrome C inhibitors 10 min, 1 h, or 5 h after 9.25 Gy whole‐body irradiation resulted in a higher survival rate in mice compared to controls [53], suggesting that targeting cytochrome C is a potential strategy for intervening in ARSs.

Exogenous apoptosis is then mainly driven by ligand activation of death receptors such as Fas, TNF‐R, and TRAIL‐R [54]. It has been reported that radiation upregulates the upregulation of Fas receptor, and ligand expression in a p53‐dependent manner in a variety of cancer cell lines with activation of caspase‐8 as well as caspase‐3 and apoptosis [55]. Caspase‐3 and caspase‐7 are common downstream and effector proteins of endogenous and exogenous apoptosis. It was found that double knockdown of caspase‐3 with caspase‐7 in mouse embryonic fibroblasts significantly reduced radiation‐induced apoptosis [56].

#### 2.2.3 Ferroptosis

Ferroptosis, as an iron‐dependent and regulated form of cell death, was first clarified in 2012 by Dixon et al. They found that a novel cell death pathway distinct from apoptosis, necroptosis, and pyroptosis, termed ferroptosis, occurs in RAS‐mutant cancer cell lines sensitive to RAS‐selective lethal compounds such as Erastin and RSL‐3 [57]. Subsequent studies have progressively revealed key aspects of the ferroptosis pathway, including the xCT system [58], GPX4 [59], ACSL4 [58], ROS accumulation [60], lipid peroxidation [58], and localization of NINJ1 at the cell membrane [61].

Lipid peroxidation and its eventual disruption of cell membrane integrity are one of the central hallmarks of ferroptosis [59]. However, radiation‐induced lipid peroxidation was observed decades before ferroptosis was identified [62]. Intracellularly accumulated iron induces ferroptosis by driving lipid ROS accumulation [63]. Multiple evidence have shown that radiation exposure significantly increases intracellular and systemic iron levels, with radiation therapy increasing serum iron levels in tumor patients [64], and astronauts with stays on the International Space Station having elevated serum iron levels [65]. Mechanistically, current studies have found that radiation promotes the release of free iron through multiple pathways, including impairing the storage capacity of ferritin by reducing iron oxides [66], causing the release of intracellular free iron by promoting hydrolysis of ferritin proteins [67] and inducing bone marrow hemorrhage [68]. In in vitro experiments, radiation has been shown to promote ferroptosis by upregulating ACSL4 and inhibiting GPX4 expression in a variety of cancer cell lines [68]. In in vivo models, malondialdehyde levels, a marker of lipid peroxidation, were significantly elevated in tissues after radiation [69]. Multiple organs of irradiated mice also showed upregulation of the expression of ferroptosis‐inducing genes such as ACSL4 and downregulation of the expression of ferroptosis‐suppressing genes such as GPX4 [69], with this trend being particularly pronounced in the small intestinal tissues. This increase in ACSL4 in conjunction with GPX4 inhibition leads to ferroptosis and lipid peroxidation in cells following radiation.

Polyunsaturated fatty acids (PUFA) enriched in phospholipids of the cell membrane system are highly susceptible to oxidation, and the concentration of PUFA in phospholipids is positively correlated with the degree of radiation‐induced lipid peroxidation [70]. ACSL4 is a key enzyme regulating the level of intracellular PUFA, and its function is to incorporate PUFA into cell membrane lipids [71]. Several studies have confirmed that radiation exposure significantly increases intracellular ACSL4 levels [72]. Thus, radiation catalyzes the peroxidation of membrane lipid PUFA by upregulating ACSL4 expression, thereby promoting ferroptosis. In vitro studies further revealed that knockdown or pharmacological inhibition of ACSL4 effectively protected cells from radiation‐induced ferroptosis [72]. In the in vivo model, treatment of mice with ACSL4 inhibitors, such as troglitazone, was sufficient to effectively alleviate radiation‐induced lipid peroxidation and tissue damage [73]. Mechanistically, radiation activates the STAT1/IRF1 signaling axis in intestinal epithelial cells, driving the upregulation of ACSL4 expression to regulate ferroptosis [74].

GPX4 plays a crucial role in maintaining cellular homeostasis. Numerous studies have shown that radiation can significantly downregulate GPX4 expression in a variety of in vitro and in vivo models [75]. GPX4 conditional knockout mice had a significantly higher mortality rate than wild‐type controls after exposure to 10 Gy of radiation, which directly demonstrates the critical radioprotective role of GPX4 [76]. Mechanistic studies suggest that radiation may inhibit GPX4 by suppressing the expression of SLC7A11, a key regulator of GPX4, a component of the xCT transport system, and by decreasing the level of glutathione, which is necessary to maintain GPX4 activity [58].

#### 2.2.4 Necroptosis

Apoptosis requires the involvement of the caspase family; however, when apoptosis is blocked via the utilization of caspase inhibitors, such as zVAD, the cell still dies. Necroptosis is the process by which cells self‐destruct when apoptosis is blocked, either through extracellular signals such as death receptor‐ligand binding or intracellular signals such as exogenous nucleic acid activation. This cellular destruction can be observed with typical manifestations resembling cellular necrosis, such as swelling of organelles, rupture of cell membranes, and breakdown of the cytoplasm and nucleus [77]. In 2005, Yuan et al. found that this necrotic‐like cellular death could be specifically inhibited by a small‐molecule inhibitor, Nec‐1 [78]. Since Nec‐1 is a specific inhibitor of the RIPK1 protein, they named the mode of death necroptosis. In recent years, it has been found that the core regulators of necroptosis include RIPK1193, RIPK3194, and MLKL195, which ultimately perform cell lysis [79].

In radiation‐induced cell death, it has been reported that Nec‐1 treatment significantly reduced radiation‐induced cell death in TPC‐1, 8505‐C, and SW13 cell lines, suggesting that radiation also induces necroptosis [80]. However, Nec‐1 failed to exert a protective effect in the H295R cells because this line lacks RIPK1. In addition to this, Nec‐1 treatment also increased cell viability in radiation‐treated human papillary thyroid cancer tissue samples. Similarly, radiation was observed to induce RIPK1, RIPK3, and MLKL phosphorylation and mediate necroptosis in cancer cells such as HIEC, MCF‐7, and MDA‐MB‐231 [81]. And it could be reversed by pharmacological addition of Nec‐1 or knockdown of MLKL expression [82]. Another study observed that exposure to 9.5 Gy radiation induced necroptosis, and the process could be blocked by Nec‐1 treatment [83].

The roles exerted by necroptosis in in vivo models remain controversial: it has been reported that application of Nec‐1 treatment to mice receiving 9.5 Gy whole‐body irradiation significantly increased their survival rates [83]. However, it has also been found that the overall survival (OS) rate of RIPK3 knockout mice after whole‐body irradiation at 6.7–7.8 Gy did not differ significantly from that of wild‐type controls [84]. These studies suggest that, in the murine model, necroptosis may not be the main type of radiation‐induced cell death [84].

#### 2.2.5 Pyroptosis

Inflammasome is a multimeric protein complex which was first discovered and named by Tschopp et al. [85]. Its core components include the cytoplasmic pattern recognition receptor as a sensor that recognizes a variety of signals, the bridging molecule ASC, and the effector protein cysteine protease caspase‐1. After assembly of the inflammasome, its primary function is to mediate the activation of caspase‐1. Activated caspase‐1 then catalyzes a series of downstream proteins, including cleavage and maturation of the proinflammatory cytokine IL‐1β and IL‐18 precursors, cleavage of the GSDMD and release of its pore‐forming active N‐terminal structural domains, which ultimately leads to a specific form of inflammatory cell death named pyroptosis [86].

The role of pyroptosis in radiation‐induced cell death has only received attention in recent years. Studies have suggested that multiple cytoplasmic sensors are involved in radiation‐induced inflammasome activation and subsequent pyroptosis. An earlier study published in 2015 by Stoecklein et al. was the first to demonstrate that IR activates the inflammasome pathway [87]. Detecting activated caspase‐1 by flow cytometry, the authors found that radiation promoted caspase‐1 activation in almost all immune cell types in the spleen on day 1 in a dose‐dependent manner. Notably, caspase‐1 activation was highly coincident with radiation‐induced cell death events, which were significantly attenuated in caspase‐1 knockout mice [87]. Further studies showed that the cytoplasmic DNA sensor AIM2 is able to sense radiation‐induced DNA damage and assemble to form AIM2 inflammasome, which in turn activates caspase‐1 and drives pyroptosis [88]. Similar to necroptosis, in vivo studies are controversial regarding the importance of pyroptosis in radiation‐induced cell death. It has been shown that AIM2, ASC, or caspase‐1 knockout mice are resistant to lethal doses of radiation. Mice with conditional knockout of caspase‐1 in intestinal epithelial cells are resistant to radioactive intestinal damage with increased survival [88]. However, Brickey et al. showed no statistically significant difference in survival in ASC, caspase‐1, and caspase‐11 knockout mouse models compared to wild‐type control mice [89]. Thus, further in vivo studies are needed to investigate the importance of pyroptosis in radiation‐induced cell death.

### 2.3 Tissue and Systemic Responses: Inflammation, Immune Modulation, and Microenvironment Remodeling

Radiation energy directly induces chemical modifications of bases (e.g., deamination or hydroxylation), resulting in base loss or mispairing [90]. Simultaneously, it causes SSBs via direct cleavage of phosphodiester bonds [91]. If adjacent sites on complementary strands are simultaneously damaged, DSBs occur—a highly lethal form of damage that is difficult to repair via HR, potentially triggering chromosomal fragmentation, rearrangements, and genomic instability [92]. Additionally, radiation induces DNA–DNA cross‐links and DNA–protein covalent cross‐links, severely impeding replication fork progression and transcriptional complex function [93]. When IR interacts with biological systems, approximately 70% of the energy is absorbed by water molecules, generating various reactive species through direct ionization and secondary reactions. These molecular injuries trigger redox imbalance (reduced GSH/GSSG ratio, thioredoxin reductase system overload) and disrupted metal homeostasis (free Fe^2^⁺ catalyzing ·OH generation via the Fenton reaction), activating the KEAP1‐NRF2 antioxidant pathway [94], NF‐κB pro‐inflammatory pathway [95], and ASK1‐JNK apoptosis pathway, forming a self‐amplifying cascade [96]. Unrepaired damage ultimately leads to cell cycle arrest, mitochondrial dysfunction, and death signaling activation, manifesting as tissue‐specific pathologies such as intestinal crypt stem cell depletion [97], alveolar epithelial barrier disruption [98], and neuronal axonal degeneration [99]. The tissue‐specific susceptibility correlates with proliferative activity and endogenous antioxidant capacity. The cascade reactions between cells triggered by radiation exposure exhibit multidimensional biological effects:

1. At the inflammatory level, damage‐associated molecular patterns (DAMPs) trigger the Toll‐like receptor (TLR) signaling pathway, inducing the release of pro‐inflammatory factors such as TNF‐α, IL‐1β, and IL‐6. These factors, in conjunction with the activation of caspase‐1 mediated by the NLRP3 inflammasome, drive pyroptosis. Meanwhile, the CXCL8/CCL2 chemokine network recruits neutrophils and monocytes for infiltration. Through the TGF‐β/Smad pathway, myofibroblasts are activated, promoting fibrosis [100].
2. In terms of immune regulation, radiation reshapes the balance between innate and adaptive immunity. Macrophages dynamically polarize toward the pro‐inflammatory M1 type (iNOS⁺) or the reparative M2 type (CD206⁺). Natural killer (NK) cells eliminate damaged cells through the perforin‐granzyme pathway [101]. In adaptive immunity, the imbalance of the CD4⁺/CD8⁺ T‐cell ratio, accompanied by the upregulation of immune checkpoints such as PD‐L1 and CTLA‐4, forms an immunosuppressive microenvironment [102].
3. In intercellular communication, the transmission of calcium waves mediated by gap junction channels (such as Cx43) induces the bystander effect. Exosomes, on the other hand, transmit damage signal molecules such as miR‐21, miR‐34a, and HSP70, enabling the transfer of radiation effects across tissues [103]. It is worth noting that low‐dose ionizing radiation (LDIR) activates adaptive responses through epigenetic reprogramming (DNA methylation/histone acetylation), including enhanced activities of SOD and CAT, DNA repair mediated by ATM and BRCA1, and mTOR‐dependent protective autophagy [104]. Its biphasic dose effect (hormesis) is manifested as enhanced insulin synthesis regulated by the p38 MAPK/PDX‐1 pathway in pancreatic β cells, without activating the ATM‐dependent DDR, which is distinct from the apoptosis induced by the excessive activation of ROS/p38 MAPK caused by high‐dose ionizing radiation (HDIR) [105].

Beyond activating classical apoptotic pathways, radiation also induces ferroptosis through iron dyshomeostasis (free Fe^2^⁺ accumulation) and lipid peroxidation [106]. This iron‐dependent cell death modality is characterized by glutathione peroxidase 4 (GPX4) activity suppression and acyl‐CoA synthetase long‐chain family member 4 (ACSL4)‐driven membrane phospholipid peroxidation—a process mechanistically distinct from DNA damage yet critically implicated in hematopoietic ARS [107]. Concurrently, radiation‐triggered sphingomyelinase activation disrupts membrane integrity, synergizing with receptor‐interacting protein kinase 3 (RIPK3)/mixed lineage kinase domain‐like pseudokinase (MLKL)‐mediated necroptosis to form an interconnected cell death network [108].

The above multi‐level reactions interact through the inflammation–immunity–metabolism axis, determining the outcome of tissue damage repair or fibrosis. Their dynamic balance is regulated by radiation dose, target cell type, and the microenvironment, providing a basis for multi‐target intervention in radiation protection and treatment strategies.

Ionizing radiation inflicts damage on biomolecules, exhibiting pathological characteristics across multiple levels. When high‐energy radiation (e.g., X‐rays or γ‐rays) penetrates biological tissues, it triggers DNA damage, inflammatory signaling networks through direct energy deposition and free radical chain reactions, leading to pathophysiological alterations and tissue injury.

In the enzymatic system, superoxide dismutase (SOD) catalyzes the conversion of superoxide anions (O_2_·^−^) into hydrogen peroxide (H_2_O_2_), and catalase (CAT) further decomposes it into H_2_O and O_2_. The glutathione system (GPx/GR/GST) maintains redox homeostasis through the GSH/GSSG cycle. In the non‐enzymatic system, vitamin E (in the lipid phase) and vitamin C (in the aqueous phase) cooperate to quench free radicals, while the thioredoxin system regulates redox signals through thiol–disulfide exchange [109].

Oxidative stress regulates cellular responses bidirectionally through the Nrf2/ARE and NF‐κB pathways: The dissociation of Keap1‐Nrf2 promotes the entry of Nrf2 into the nucleus, where it binds to the antioxidant response element (ARE) and upregulates detoxifying enzymes such as heme oxygenase‐1 and NADPH quinone oxidoreductase 1 (NQO1). On the other hand, the activation of NF‐κB mediated by IκB kinase (IKK) determines cell fate through the dynamic balance between pro‐inflammatory factors (such as the NLRP3 inflammasome) and pro‐survival genes.

Mitochondria, as the core hub of oxidative stress, exhibit dysfunction manifested as increased electron leakage in electron transport chain (ETC) complexes I/III, collapse of the membrane potential (ΔΨm), and abnormal opening of the mitochondrial permeability transition pore (mPTP). This leads to the release of cytochrome c and apoptosis‐inducing factors (AIF), activating both caspase‐dependent and caspase‐independent apoptosis pathways.

Notably, IR promotes the leakage of mitochondrial DNA (mtDNA) into the cytoplasm through the voltage‐dependent anion channel (VDAC), activating the cGAS–STING innate immune signaling axis, triggering a type I interferon response, and exacerbating hematopoietic injury [110]. Experiments have confirmed that the VDAC1 inhibitor DIDS and cGAS inhibitors can alleviate radiation‐induced depletion of bone marrow stem cells and abnormal infiltration of macrophages (F4/80⁺), providing a new strategy for the prevention and treatment of ARS by targeting the mitochondria–cGAS pathway [111].

Ionizing radiation also reshapes the tumor microenvironment (TME) through coordinated changes in vasculature, extracellular matrix (ECM), stromal fibroblasts, and immune checkpoints. Vascular injury and perfusion heterogeneity generate and maintain hypoxia, stabilizing HIF‐1α and transcriptional programs that upregulate VEGF and CXCL12, thereby promoting angiogenic rebound and recruitment of CXCR4⁺ bone‐marrow–derived cells after RT—processes linked to radioresistance and tumor regrowth [112]. In parallel, IR drives ECM remodeling and stiffness through lysyl‐oxidase–mediated collagen cross‐linking and altered matrix turnover, which enhances invasive signaling and impedes drug/immune‐cell penetration; therapy‐induced ECM reprogramming is increasingly recognized as a determinant of response and relapse [113].

Stromal fibroblasts are similarly reprogrammed: RT activates and expands cancer‐associated fibroblasts (CAFs), which secrete TGF‐β, IL‐6, CXCL12, and MMPs, fostering fibrosis, epithelial–mesenchymal transition, metabolic coupling, and immune exclusion—altogether shaping a pro‐survival niche for irradiated tumors [114]. On the immune axis, IR has a dual face: cGAS–STING–dependent type I interferon can promote antitumor priming, yet PD‐L1 is frequently upregulated on tumor and myeloid populations post‐RT, reinforcing local immunosuppression and selection for exhaustion phenotypes; these dynamics motivate combinations with checkpoint blockade and chemokine‐axis modulators (e.g., CXCL12/CXCR4 inhibitors) to counteract maladaptive remodeling [115]. Collectively, these vascular–matrix–stromal–immune couplings explain how IR can steer tissues toward either repair or fibrosis and highlight actionable nodes for radiosensitization and normal‐tissue protection within the remodeled microenvironment.

This cascade mechanism reveals that mitochondria are not only effectors of oxidative stress but also key mediators of non‐targeted radiation effects. Its regulatory network provides a basis for multi‐target intervention in radiation protection.

## 3 Radiation Biodosimetry: For Nuclear Emergency Response

Major radiation incidents demand rapid, scalable, and clinically actionable assessment of absorbed dose to support triage and guide treatment. Biodosimetry must operate within tight time windows, handle large populations, and balance accuracy with throughput, portability, and cost. Practical constraints include partial‐body versus total‐body exposure, mixed radiation fields, dose‐rate effects, and confounders such as burns, trauma, age, sex, and comorbidities. Accordingly, an “ideal” biodosimetric solution would combine low limits of detection, automation, high throughput, and robust performance across heterogeneous cohorts. This section surveys the measurement landscape from established cytogenetic assays and DNA damage–response proxies to omics‐based biomarkers spanning transcriptomes, proteomes, metabolomes/lipidomes, and microbiomes, sampled from accessible matrices. We emphasize the core trade‐off—gold‐standard specificity versus operational speed—and the growing role of machine learning for automated image scoring, signature selection, and dose prediction. Where relevant, we note species and matrix differences, retrospective windows, and the need for multi‐model, cross‐cohort validation.

### 3.1 Role of Biodosimetry in Triage and Medical Management

During a major radiation event, a large number of people need to be rapidly assessed for radiation damage to ensure effective medical treatment and appropriate use of medical resources [116]. Accurate and rapid radiation dose assessment play a crucial role in diagnosis and guiding treatment of acute radiation sickness. Ionizing radiation can directly cause DNA damage, chromosomal aberrations, protein destruction or alterations, and modifications in metabolites. Existing research, through screening in genomics, transcriptomics, metabolomics, lipidomics, proteomics, and microbiomics, has identified several radiation‐sensitive biomarkers that hold potential as biological indicators for estimating radiation dose, assessing tissue damage, and aiding medical stratification [117].

According to a study by the U.S. government, detonating a 10‐kiloton improvised nuclear device (IND) in a large city like Washington would result in over 300,000 people suffering radiation‐related injuries, with over 50% of them recovering without any medical intervention, but even with medical intervention, approximately 50,000 people might still die [118]. When exposed to 1–2 Gy of whole‐body radiation, symptoms such as dizziness, fatigue, insomnia, and decreased appetite may occur [119]. When exposed to 2–6 Gy, these symptoms worsen, and damage to the hematopoietic system begins to manifest, characterized by a continuous decrease in white blood cell and platelet counts, although recovery is generally possible [120]. With exposure above 6 Gy, symptoms appear earlier and progress more rapidly [121, 122], (p. 199). When the dose exceeds 10 Gy, severe damage to intestinal epithelial cells occurs, primarily manifesting as gastrointestinal symptoms such as abdominal pain, vomiting, and bloody stools [123]. At doses above 50 Gy, neurological symptoms arise, including ataxia, tonic‐clonic seizures, opisthotonos, and disorientation [124, 125]. The key to treating RITI is to initiate medical intervention as early as possible. For patients exposed to doses less than 2 Gy, symptomatic treatment is sufficient [126]. Patients exposed to doses between 2 and 6 Gy require prompt hospitalization and targeted treatment for potentially life‐threatening complications. For those exposed to doses greater than 6 Gy, early evaluation and preparation for hematopoietic stem cell transplantation are essential [119, 127].

Currently, the most commonly used models for studying radiation biodosimeters are experimental animal irradiation models and in vitro irradiation of cultured blood samples collected from healthy donors [128, 129]. Although these two approaches cannot perfectly simulate human conditions, analyzing the changes occurring both in vitro and in vivo, as well as the similarities and differences between animal and human responses, enables reasonable interpretation of related phenomena. This makes the development of biological radiation dosimeters feasible for practical human applications. An ideal radiation dosimeter should exhibit characteristics such as rapid response, high throughput, automation, cost‐effectiveness, and the capacity to detect radiation at lower doses (less than 0.5 Gy) [130]

At present, the main methods for determining radiation doses are the micronucleus assay and the DCA [131, 132, 133]. Both methods require significant time and manpower to perform and are difficult to apply to certain exposed populations. In a scenario involving large‐scale radiation casualties, the required method should balance accuracy and speed, making traditional methods difficult to apply [134]. DCA is considered the “gold standard” in biological radiation dose assessment due to its ability to accurately calculate radiation doses [132]. It remains difficult to replace in practical work, and current efforts are focused on using machine learning to automatically interpret DCA results, which could reduce the time required for analysis [135]. However, the experiment faces several issues: (1) Different organs and tissues have varying radiation sensitivities and absorption rates. (2) The repair of aberrant chromosomes by the body can affect dose estimation. (3) Aberrant lymphocytes may disappear relatively quickly, limiting retrospective dose estimation. Therefore, blood can only be collected a few hours before the onset of leukopenia events, and peripheral blood lymphocytes need to be cultured for 48 h to conduct DCA testing [136, 137]. Ideally, interventions and countermeasures should be employed before the onset of ARS or chronic radiation sickness [138].

We will elaborate in the following sections. The article mainly reviews existing assessment methods, including biomarkers from transcriptomics, metabolomics, lipidomics, proteomics, and microbiomes, as well as biological radiation dose detection devices and their development‐related content.

### 3.2 Omics‐Based Biodosimetry Approaches: Transcriptomics, Metabolomics, Proteomics

#### 3.2.1 Transcriptomics

RNA biomarkers have been widely used to predict the prognosis of various diseases [139, 140]. In the field of biodosimetry, RNA markers can also be used to assess human radiation exposure [141, 142]. The radiation dose assessment method based on RNA biomarkers has many advantages compared to commonly accepted dose assessment methods: (1) It does not require excessive manual labor and has a simple process. (2) It mainly relies on machine‐based batch testing. (3) RNA can be directly extracted from whole blood without the need for lymphocyte culture, significantly reducing the detection time [143].

Another advantage of using RNA molecules as biomarkers is their relative stability. The RNA in whole blood primarily consists of extracellular RNA and intracellular RNA. Intracellular RNA mainly refers to RNA in leukocytes and mature erythrocytes. In leukocytes (such as lymphocytes), the half‐life of mRNA under resting conditions is approximately 2–4 h [144]. rRNA and tRNA are highly stable, with half‐lives that can extend up to several days [145]. In mature erythrocytes, the half‐life of mRNA is about 8–12 h, while that of miRNA is approximately 24 h [146].

Researchers have found that in whole blood samples incubated at room temperature, the half‐lives of mRNA, microRNA (miRNA), and long non‐coding RNA (lncRNA) were 16.4, 16.4, and 17.5 h, respectively. Another advantage of using RNA molecules as biomarkers is their relative stability: in whole blood samples incubated at room temperature, the half‐lives of mRNA, miRNA, and lncRNA are 16.4, 16.4, and 17.5 h, respectively [117, 147].

The role of mRNA in radiation response through DNA repair, inflammation, and other pathways is direct because radiation damages cells and mRNA responds to this damage [77, 148, 149]. Researchers selected radiation‐sensitive genes in mice by RNA sequencing and evaluated their utility as radiation biodosimeters in human cell lines. They identified five genes with significantly differential expressions after radiation exposure and they found that positive cofactor 4 (PC4) had a good correlation with radiation dose in human lymphoblastoid cell line after irradiation. The relative expression of PC4 gene showed a good linear correlation with the radiation dose after 1–5 Gy irradiation. In addition, PC4 gene can also be rapidly recruited to the DNA damage sites [150].

In‐depth investigation reveals that this characteristic may be related to the extensive involvement of PC4 in various key intracellular mechanisms, such as DNA replication, damage repair, chromatin construction, and cell cycle regulation [151].

A study identified 74 characteristic genes that changed after irradiation, with over one‐third of these genes being regulated by TP53 [152]. Among them, CDKN1A and GADD45A show good specificity in both low and medium‐high doses of radiation [153, 154]. Subsequent research has also demonstrated that CDKN1A and GADD45A exhibit good radiation dose characteristics in mice [154], NHPs [155], and human cell lines [156], making them promising candidates for effective radiation biomarkers. A team conducted transcriptome sequencing on blood samples from 11 healthy volunteers, 13 severely burned patients, and 37 severely traumatized patients, identifying a total of 12 radiation‐specific genes unrelated to trauma and burns; ROC curve analysis shows that DDB2 and MDM2 perform the best in diagnosing radiation types and evaluating radiation doses [157]. What is more, another research groups identified that the CCNG1 and CDKN1A mRNA performed optimally in radiation dose response, independent of trauma, burns, age, and sex. Additionally, the CCNG1 protein revealed a strong linear correlation between radiation dose and time post‐irradiation [158].

However, we cannot overlook the roles of ncRNAs in promoting repair or long‐term functional impairment, and miRNAs are small (17–25 nucleotides) ncRNAs that regulate protein and mRNA expression by binding target mRNAs and inducing degradation [159]. In contrast, lncRNAs are longer than 200 nucleotides and modify transcription and translation by altering chromatin regulation, RNA stability, and protein binding within the nucleus [160]. Both types of ncRNAs have been proposed as biomarkers for cancer, heart dysfunction, and various neurological disorders [161, 162, 163]. Several studies have previously reported the emerging roles of miRNAs and lncRNAs as potential biomarkers for radiation‐induced injuries in mouse and non‐human primate (NHP) models [164, 165, 166]. Researchers administered different doses of radiation to mice and observed acute changes in biological pathways using ingenuity pathway analysis (IPA) 48 h after exposure to different radiation doses, identifying potential RNA biomarkers for predicting radiation injury [164]. Another study compared levels of over 600 miRNAs in mouse serum using the nanoString Technologies nCounter multiplex platform at radiation doses ranging from 1 to 12 Gy and time points of 24 and 48 h. They identified several sensitive miRNA markers for lymphocyte depletion and bone marrow injury, demonstrating the potential use of miRNA changes as an indicator for radiation accidents and for toxicity and response assessments during and after therapeutic radiation using minimally invasive methods [167]. Researchers reported an expandable radiation biodosimetry assay based on dual microRNAs (miR‐RAD), which accurately estimated absorbed IR doses through mathematical modeling of changes in miR‐150‐5p/miR‐23a‐3p in whole blood, achieving a screening sensitivity of 95.9% for subjects exposed to 2 Gy radiation, indicating high predictability for absorbed doses above 2 Gy, with a specificity of 89% (160/180), but lower sensitivity at doses greater than 6 Gy at 55% (33/60). However, there is a lack of dose–response analysis after mixed neutron and gamma radiation exposures, and possible changes in dose effects from low‐dose‐rate radiation compared to high‐dose‐rate radiation‐induced injuries [168]. Therefore, further refinement and calibration are needed for radiation biodosimetry using miR‐150‐5p and miR‐23a‐3p. Ionizing radiation also affects the expression of circular RNAs (circRNAs) in healthy human cell lines and tumor tissues [169].

As a category of ncRNA, circRNAs are primarily formed through a back‐splicing mechanism. This unique biogenesis process can be induced by various factors, including protein dimerization, sequence complementarity of flanking introns, exon‐skipping mechanisms, and intron debranching [170]. Relevant studies demonstrate that under IR conditions, certain circRNAs exhibit prolonged induced expression patterns in irradiated mouse embryonic brains, primary mouse cortical neurons, and murine blood samples [169].

Researchers screened for differentially expressed circRNAs in the irradiated human lymphoblast‐like cell line AHH‐1 and validated these at various doses. A total of 11 radiation‐induced differentially expressed circRNAs were identified and validated, which can distinguish different doses and exposure conditions within 48 h [171].

In addition, researchers have discovered a novel RNA N6‐methyladenosine (m6A) modification‐based radiation dose biomarker and established a model for predicting human radiation exposure based on this modification. The study conducted genome‐wide screening of mRNA molecules in peripheral blood mononuclear cells (PBMCs) responsive to IR in mice, revealing significant changes in RNA m6A levels after IR exposure. Of note, RNA m6A levels of the Ncoa4 (nuclear receptor coactivator 4) gene remained significantly elevated up to 28 days after radiation exposure, indicating its excellent performance as an ideal radiation biomarker and further ruling out the impact of inflammation, gender, and age on dose–response relationships, demonstrating its specificity to IR responses [172]. What is more, RNA microarray analysis [173, 174] and next‐generation sequencing (NGS) [175] are also used to predict radiation doses within the organism.

Although these studies can all reflect the magnitude of radiation received by the human body under specific conditions, when using RNA as a radiation biodosimeter in practical applications, it is necessary to consider the situation caused by individual differences, and to exclude non‐specific transcriptomic changes brought about by factors such as gender [176], age [177], and environment [178]. Currently, there is also a lack of multi‐model validation for transcriptomic mRNA radiation biodosimeters. Identifying stable and reliable mRNA candidates and validating them across multiple models are key research highlights in establishing such biodosimeters. The radiation‐sensitive mRNA and miRNA biomarkers under different doses and models are described in Tables 1 and 2, respectively.

**TABLE 1:** Summary of radiation‐sensitive mRNA biomarkers in different doses and models.

Gene	Full name	Effect	Range (Gy)	Method	Species	Sample type	Types	Refs.
GDF15	Growth differentiation factor 15	Cell‐cycle arrest	0–12	q‐PCR	Mouse	Heart	X‐ray	[173, 176]
CKAP2	Cytoskeleton‐associated protein	Cell‐cycle arrest	0–12	q‐PCR	Mouse	Heart	X‐ray	[173]
CCNG1	Cyclin G1	Enabling protein binding	1, 2, 3, 4, 6, 8	q‐PCR	Mouse	Blood	X‐ray	[179]
AEN	Apoptosis enhancing nuclease	Enabling DNA exonuclease activity	1, 2, 3, 4, 6, 8	q‐PCR	Mouse	Blood	X‐ray	[179, 180, 181]
PHLDA3	Pleckstrin homology like domain family A member 3	Enabling phosphatidylinositol‐3,4,5‐trisphosphate binding	1, 2, 3, 4, 6, 8	q‐PCR	Mouse	Blood	X‐ray	[179]
CCNA2	Cyclin A2	G1/S and G2/M cell cycle regulators	0–10	q‐PCR	Mouse	Blood	γ‐ray	[182]
DDA3	Differential display and activated by P53	Suppressing cell growth	50, 200, 1000 cGy	q‐PCR	Mouse	Blood	γ‐ray	[183]
CDKN1A	Cyclin‐dependent kinase inhibitor 1A	Enabling cyclin binding	0, 0.5, 2, 5, 8	q‐PCR	Mouse, human	Heart, blood	X‐ray,γ‐ray	[173, 184]
CD117	KIT proto‐oncogene, receptor tyrosine kinase	Regulation of cell survival and proliferation	2.5, 5	q‐PCR	Baboons	Blood	X‐ray	[185]
VSIG4	V‐Set and immunoglobulin domain containing 4	Negative regulator of T‐cell proliferation	2.5, 5	q‐PCR	Baboons	Blood	γ‐ray	[185]
GBP2	Guanylate binding protein 2	Oxidative killing and antiviral activity	2.5, 5	q‐PCR	Baboons	Blood	γ‐ray	[111, 185]
GLUL	Glutamate‐ammonia ligase	Synthesis of glutamine	2.5, 5	q‐PCR	Baboons	Blood	γ‐ray	[188]
PCNA	Proliferating cell nuclear antigen	RAD6‐dependent DNA repair pathway	0, 0.5, 2, 5, 8	q‐PCR	Human	Blood	γ‐ray	[176, 189]
DDB2	Damage‐specific DNA binding protein 2	Facilitating the cellular response to DNA damage	0, 0.5, 2, 5, 8	q‐PCR	Human	Blood	γ‐ray	[176, 189]
PHPT1	Phosphohistidine phosphatase 1	Regulating CD4 T lymphocytes by dephosphorylation and inhibition of KCa3.1 channels	0, 0.5, 2, 5, 8	q‐PCR	Human	Blood	γ‐ray	[176, 189]
FDXR	Ferredoxin reductase	Encoding a mitochondrial flavoprotein	0–4	q‐PCR	Human	Blood	X‐ray	[112]
POU2AF1	POU class 2 homeobox associating factor 1	Enabling transcription coactivator activity	0, 0.5, 4, 5	q‐PCR	Human	Blood	X‐ray	[190, 191]
WNT3	Wnt family member 3	Activation of the WNT‐beta‐catenin‐TCF signaling pathway	0, 0.5, 5	q‐PCR	Human	Blood	X‐ray	[190]
GADD45A	Growth arrest and DNA damage inducible alpha	Activation of the p38/JNK pathway	2, 50 cGy	q‐PCR	Human	ML‐1	γ‐ray	[191, 192]
MDM2	MDM2 proto‐oncogene	Promoting tumor formation	2, 50 cGy	q‐PCR	Human	ML‐1	γ‐ray	[153, 191, 192]
XPC	XPC complex subunit	Early steps of global genome nucleotide excision repair	0.2, 0.5, 1, 2	q‐PCR	Human	Blood	γ‐ray	[193]
NOCA4 m6A	N6‐methyladenosine of NOCA4	Encoding an androgen receptor coactivator	0, 0.2, 0.5, 1, 2, 4, 6	q‐PCR	Human	Blood	X‐ray	[172]

**TABLE 2:** Summary of radiation‐sensitive miRNA biomarkers in different doses and models.

Gene	Effect	Range (Gy)	Method	Species	Sample Type	Types	Refs.
miR‐187‐3p	Predicting radiation‐induced hematopoietic damage	0, 6.5, 8	q‐PCR	Mouse	Blood	γ‐ray	[186]
miR‐27a‐3p	Predicting radiation‐induced hematopoietic damage	0, 6.5, 8	q‐PCR	Mouse	Blood	γ‐ray	[186]
miR‐30a‐3p	Predicting radiation‐induced hematopoietic damage	0, 6.5, 8	q‐PCR	Mouse	Blood	γ‐ray	[186]
miR‐30c‐5p	Predicting radiation‐induced hematopoietic damage	0, 6.5, 8	q‐PCR	Mouse	Blood	γ‐ray	[186]
miR‐223–3p	Response to different radiation doses at 6, 24, and 48 h	0, 2, 4, 8, 12, 15	q‐PCR	Mouse	Blood	X‐ray	[194]
miR‐151‐3p	Dose‐specific biomarkers of 8.0 Gy IR exposure	2, 6.5, 8	q‐PCR	Mouse	Blood	X‐ray	[195]
miR‐128‐3p	Dose‐specific biomarkers of 8.0 Gy IR exposure	2, 6.5, 8	q‐PCR	Mouse	Blood	X‐ray	[195]
let‐7a‐5p	Upregulated after radiation	2, 3, 4	nanoString	Mouse	Blood	X‐ray	[196]
miR‐188‐5p	Downregulation after radiation	2, 3, 4	nanoString	Mouse	Blood	X‐ray	[196]
miR‐340‐5p	Predicting high‐dose and high‐risk radiation exposure	0, 1, 2, 4, 8	q‐PCR	Mouse	Blood	X‐ray	[197]
miR‐574‐3p	Predicting complete blood cell reduction	0, 2.5, 5	q‐PCR	Baboons	Blood	γ‐ray	[198]
miR‐342‐3p	The best candidate for prediction of HARS 2–3°	0, 2.5, 5	q‐PCR	Baboons	Blood	γ‐ray	[198]
miR‐16‐2	Associating with radiation survival	0, 7.2	q‐PCR	Baboons	Blood	γ‐ray	[198]
miR‐574‐5p	Early biological response markers with clear dose–response detectable within 24 h	1, 3, 6.5	q‐PCR	NHP	Blood	γ‐ray	[199]
miR‐150‐5p/ miR‐23a‐3p	Radiation dose reconstruction	0.5–3.5	q‐PCR	Human	Blood	Neutrons,γ‐ray	[143]
miR‐1228‐3p	Negative correlation with gene expression of UBE2D 2 and PPP2R2D	10, 100, 1000 mGy	q‐PCR	Human	CGL1	X‐ray	[200]
miR‐758‐5p	Reduced expression at doses of 10 mGy and 100 mGy	10, 100, 1000 mGy	q‐PCR	Human	CGL1	X‐ray	[200]
miR‐146a‐5p	Grouping dose classes in five steps	0,1,2,4, 8, 10	q‐PCR	Human	PHAOEC	X‐ray	[201]
miR‐181a‐5p	Grouping dose classes in five steps	0,1,2,4,8, 10	q‐PCR	Human	PHAOEC	X‐ray	[201]
miR‐3529‐3p	Grouping dose classes in five steps	0, 1, 2, 4, 8, 10	q‐PCR	Human	PHAOEC	X‐ray	[201]

#### 3.2.2 Metabolomics

Metabolites have achieved significant success in predicting diseases (predicting cancer, among other diseases) and have also received widespread attention in predicting radiation doses. Common samples include saliva, tissues, blood, or urine from post‐radiation patients [179]. The two main analytical techniques used to measure metabolomic changes are mass spectrometry [180] and nuclear magnetic resonance (NMR) [181]. Nuclear magnetic resonance relies on the inherent magnetism of specific atoms within metabolites, allowing for quantitative and qualitative measurements through the measurement of chemical shifts after electromagnetic pulses. Mass spectrometry separates and quantifies small metabolites from body fluids or tissues based on molecular mass and ionization [182]. Nuclear magnetic resonance spectroscopy is robust, reproducible, unbiased, and quantitative, provides information at the molecular structural level, requires minimal sample preparation, and involves minimal data processing. It is well suited for measuring large samples, multiple sites, and longitudinal studies [183]. However, gas chromatography‐mass spectrometry (GC‐MS) and liquid chromatography‐mass spectrometry (LC‐MS) have the advantage of being more sensitive than NMR, with detection limits typically 10–100 times higher than NMR. Both methods are high‐throughput and require data processing and statistical analysis to provide useful information. Currently, these two technologies and sciences are gradually being integrated into applications to enhance practicality and overcome inherent limitations in metabolomics applications [184].

In previous studies, various potential metabolomic biomarkers have been proposed [185]. For example, tissue polypeptide antigen (TPA) can serve as an indicator of salivary gland radiation injury [186], Flt3 ligand is associated with radiation‐induced bone marrow injury [187], and glutamine predicts fatal ARS of the gastrointestinal tract within 21 days after exposure to 12 Gy of radiation [188].

Products from the linoleic acid metabolism pathway and glycerophospholipid metabolism pathway can predict radiation dose levels in the 0–5 Gy range [189]. Metabolites have the function of distinguishing radiation doses at relatively early time points (within 24 h).

Researchers revealed through animal experiments the biological phenomenon of IR‐induced reprogramming of intestinal amino acid metabolism. Twenty‐four hours after exposure to 2 Gy and 20 Gy doses, eight amino acids—serine, isoleucine, leucine, methionine, valine, threonine, alanine, and proline—in mouse intestinal tissues showed significant dose‐dependent elevation, with their changes positively correlated to histopathological damage such as intestinal villus atrophy and crypt structure disruption [190].

Studies suggest this phenomenon may arise from IR triggering ROS‐mediated oxidative stress, activating autophagy signaling pathways to enhance intracellular protein catabolism, leading to free amino acid accumulation [191, 192]. These elevated amino acids may exert their biological effects through a dual mechanism: on the one hand, by modulating the NF‐κB signaling pathway to suppress inflammatory responses in intestinal epithelial cells [193]; on the other hand, by serving as precursors for certain amino acids, such as alanine and leucine, to participate in gluconeogenesis or ketogenesis, thereby providing energy metabolism support for radiation‐induced damage repair. This suggests that they may constitute an adaptive protective mechanism in response to radiation stress [194, 195, 196].

Based on these findings, researchers propose that intestinal amino acid metabolic profiles (particularly branched‐chain and sulfur‐containing amino acids) could serve as novel biomarker combinations for evaluating gastrointestinal radiation injury [197], though key regulatory nodes in the autophagy‐amino acid metabolism axis and their interaction mechanisms with DNA damage repair networks require further elucidation through proteomics and gene editing technologies [198]. Metabolomic analyses of biofluids such as saliva, urine, and serum offer diversified strategies for radiation exposure assessment.

In mouse models, salivary metabolomics identified eight metabolites—3‐hydroxydecanoic acid, L‐phenylalanine, deoxyadenosine monophosphate (dAMP), and others—that could significantly distinguish 0.5, 3, and 8 Gy doses within 7 days post‐irradiation, with their dynamic changes positively correlated to DNA damage severity [199]. Additionally, urinary metabolites such as N6,N6,N6‐trimethyllysine and taurine were found to stably differentiate total body irradiation (TBI) and partial body irradiation (PBI) exposures at 0–8 Gy, with minimal interference from gender or shielding effects [200, 201]. NHP serum metabolomics further validated that integrated lipid‐amino acid multi‐omics models significantly reduce dose estimation errors [202]. However, the molecular regulatory mechanisms underlying these metabolomic alterations, particularly the dynamic interaction networks between radiation–responsive metabolites and processes like DNA damage repair and cell fate determination, require further investigation using spatiotemporal‐resolved metabolic flux analysis and single‐cell metabolomics technologies. The description of radiation‐sensitive metabolic biomarkers under different doses and models is presented in Table 3.

**TABLE 3:** Summary of radiation‐sensitive metabolites biomarkers in different doses and models.

Metabolite	Range (Gy)	Method	Species	Sample Type	Types	Refs.
Phenylpyruvate	0, 5, 12	LC/MS	Mouse	Fecal	γ‐ray	[258]
Taurocholic acid	0, 5, 12	LC/MS	Mouse	Fecal	γ‐ray	[258]
Citric acid	0, 3, 6, 7, 8, 11	UPLC‐TOFMS	Mouse	Urine	γ‐ray	[223]
Creatine	0, 8	NMR	Mouse	Urine	X‐ray	[259]
Taurine	0, 8	NMR	Mouse	Urine	X‐ray	[223, 259]
Carnitine	3, 8, 15	LCMS	Mouse	Urine	γ‐ray	[260]
Xanthine	0, 8	NMR	Mouse	Urine	γ‐ray	[223]
Isethionic acid	0, 8, 15	LC/MS	Mouse	Urine	γ‐ray	[260]
2'Deoxyxanthine	0, 3	GCMS	Mouse	Urine	γ‐ray	[261]
Hexosylceramides	0, 3	LC/MS	Mouse	Urine, Blood	X‐ray	[202]
Sphingomyelin	0, 3	LC/MS	Mouse	Urine, Blood	X‐ray	[202]
LPE	0, 3	LC/MS	Mouse	Urine, Blood	X‐ray	[202]
Creatinine	8, 8.72	FIA‐LC	Mouse	Blood	γ‐ray	[262]
HIST1H1C	0, 2, 3.5, 8	ELISA	Mouse	Blood	γ‐ray	[245]
Omega‐6/Omega‐3	0, 8	ACQUITY UPLC	Mouse	Blood	γ‐ray	[256]
Threonine	0, 8, 10, 12, 14	FIA‐LC	Mouse	Small intestine, Plasma	X‐ray	[263]
Xanthine	0, 2, 4, 6, 7	SID‐SPE‐DMS‐MS	NHP	Urine	γ‐ray	[264]
N‐Acetyltaurine	1, 3.5, 6.5, 8.5	UPLC‐QTOF	NHP	Urine	γ‐ray	[265]
Lactic acid	0, 2, 4, 6, 7, 10	GC‐TOF‐MS	NHP	Blood, Urine	γ‐ray	[266]
L‐Carnitine	0, 2, 4, 6, 7, 10	UPLC‐QTOF	NHP	Blood	γ‐ray	[250]
Taurine	0, 2, 6.5, 7.2	UPLC‐QTOF	NHP	Blood	γ‐ray	[225]
SRC	0, 4	TMT	NHP	Blood	γ‐ray	[243]
TGF β	0, 4	TMT	NHP	Blood	γ‐ray	[243]
FN 1	0, 4	TMT	NHP	Blood	γ‐ray	[243]
Valine	0, 2, 4, 6, 7, 10	LC/MS	NHP	Blood	γ‐ray	[250]
Proline	0, 2, 4, 6, 7, 10	LC/MS	NHP	Blood	γ‐ray	[250]
Xanthine	0, 1.25, 2.5, 3.75	TOFMS	Human	Urine	γ‐ray	[267]
Uric acid	0, 1.25, 2.5, 3.75	TOFMS	Human	Urine	γ‐ray	[267]
Butyryl‐coenzyme A	35–66 Gy local radiotherapy	LC‐MS/MS	Human	Urine	γ‐ray	[268]
2‐Ketohexanoic acid	35–66 Gy local radiotherapy	LC‐MS/MS	Human	Urine	γ‐ray	[268]
γ‐H2AX	0, 0.5, 1, 2, 4, 8	Confocal microscopy	Human	NHDFs	γ‐ray	[269]

#### 3.2.3 Proteomics

Like metabolomic analysis, proteomic analysis can also be used to measure the harmful effects of radiation exposure. Radiation may alter regulatory networks or induce protein modifications such as decarboxylation, disulfide bonds, or aggregation, subsequently affecting the steady‐state levels of specific proteins [203, 204].

**FIGURE 2:**
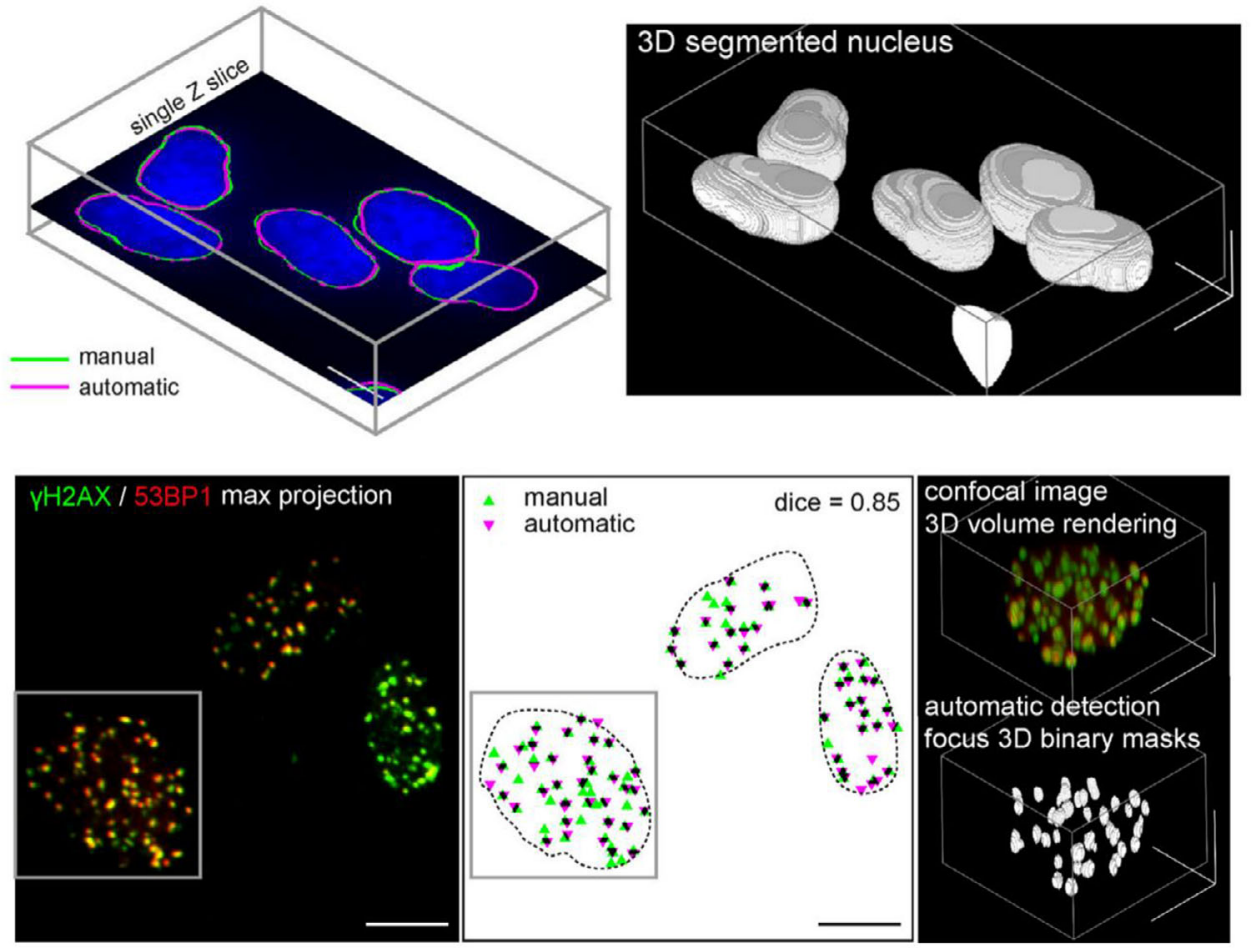
Artificial intelligence image recognition software: Identifying the number of radiation‐induced damage foci by detecting 3D confocal multichannel data. The accuracy of automated IRIF detection was compared to that of manual annotation performed by two experienced experts on the maximum‐projection images. An example of automated IRIF detection and segmentation is presented in Figure 2.

γ‐H2AX is a classic biomarker for DSBs, where H2AX (a variant of histone H2A) replaces traditional H2A in nucleosomes. Following DNA DSB damage, the Ser139 site on H2AX undergoes rapid phosphorylation, forming γ‐H2AX [205]. This phosphorylation is a crucial component of the DDR, typically occurring within minutes after DNA damage, followed by the recruitment of other signaling factors such as MDC1 and 53BP1 into the DNA repair cascade [206]. Therefore, γ‐H2AX offers a highly superior method for assessing radiation doses, as the quantity of γ‐H2AX can estimate the level of radiation exposure within the body. Studies have demonstrated its detectability in various cell types and tissue slices including cell lines [207], peripheral blood lymphocytes [21, 208], splenocytes [209], bone marrow cells [210], oral cells [211], hair [212], and xenografts [213]. Typically, four methods are employed to measure γ‐H2AX levels in cells exposed to IR: quantitative measurement via microscopy (automated or visual) [214], standard western blotting techniques [213], qualitative analysis through flow cytometry based on fluorescence intensity [21, 215], and enzyme‐linked immunosorbent assay (ELISA) [208]. The development of imaging flow cytometry has improved the detection of γ‐H2AX. Imaging flow cytometry, a relatively newer technology, combines the speed of flow cytometry with the imaging capabilities of traditional microscopy. It can acquire cell images at high speeds from suspended samples, up to 1000 cells/second, faster than automated microscopy systems, and without the need for high‐quality glass slides [216]. Cell images produced by imaging flow cytometry are much more complex compared to traditional flow cytometry data [217]. Researchers demonstrated through imaging flow cytometry of healthy human peripheral blood at 0.5, 1, 3, 6 and 24 h post‐irradiation, that within 1‐h post‐irradiation, the fluorescence intensity of γ‐H2AX showed a linear correlation with radiation dose (*R* ^2^ = 0.98). Additionally, at other time points, the fluorescence intensity of γ‐H2AX was significantly superior to the dose response of γ‐H2AX foci per cell, which may be due to cells exposed to higher doses of radiation producing numerous closely spaced γ‐H2AX foci, making accurate identification challenging in low‐resolution images [218]. The rapid development of artificial intelligence has also facilitated the development of dosimetry. Researchers also reconstructed three‐dimensional images of irradiated cells and employed a deep learning‐based fully automatic algorithm for counting and morphological analysis of ionizing radiation‐induced foci (IRIFs), enhancing the quantification of DSBs. Compared to previously published methods, this algorithm is applicable to 3D confocal multichannel data rather than single channel 2D slices or maximum image projections. It addresses the challenge of accurate identification of adjacent foci merging in low‐resolution images in traditional fluorescent staining of γ‐H2AX^268^ (Figure 2). Overall, γ‐H2AX detection greatly reduces analysis time and offers higher sensitivity compared to DCA and micronucleus assay (MN). The γ‐H2AX assay may become an attractive choice in the development of radiation biodosimeter and related emergency triage tools.

However, its high metabolic rate and poor retrospective ability significantly limit its application. Immunofluorescence results from a mouse radiation model show that on day 1 or later after irradiation, an increase in γ‐H2AX foci is almost undetectable at doses ≤ 1 Gy. Even at an irradiation dose of 6.5 Gy, γ‐H2AX foci are still undetectable on day 7 post‐irradiation [172].Therefore, considering the application conditions of radiation dosimeters in special battlefield environments, it is essential to have the ability to maintain long‐term stability. Researchers exposed rats to single doses of 0, 1, 3, and 5 Gy of γ‐ray whole‐body irradiation and found that differentially expressed proteins in the plasma after radiation exposure were primarily related to immune response, phagocytosis, and signal transduction following IR. The expression levels of candidate radiation‐sensitive protein biomarkers were confirmed using enzyme‐linked immunosorbent assay. The researchers discovered that within 7 days after radiation exposure, changes in the expression of alpha‐2‐macroglobulin (A2m), chromogranin‐A (CHGA), and glutathione peroxidase 3 (GPX3) may have potential as biomarkers for assessing radiation exposure [242]. In a study involving protein differential expression after NHP irradiation, eight rhesus monkeys (four males and four females) were subjected to semi‐lethal dose (4 Gy) whole‐body irradiation. Blood samples were collected on day 6 and day 28 post‐irradiation for proteomic analysis. The findings confirmed that under 4 Gy whole‐body irradiation, NHP whole‐blood proteomic changes were associated with MAPK signaling pathways. Protein SRC, TGF‐β, and NFATC2 were immediately induced one day after irradiation, resulting in increased transcriptional activity. These changes persisted for up to 1‐month post‐exposure and were consistent with blood cell damage, death, and regeneration [220].

The method of sample collection is crucial for screening work in battlefield environments. Researchers irradiated NHPs with 6.7 Gy and 7.4 Gy whole‐body radiation and characterized the proteomics of blood and urine. They found significant differences in 10 proteins in animal plasma and urine between doses and time points. These proteins include serum amyloid protein A, uncharacterized proteins F7GRY2 (C9), and F7DHQ1 (CRP), which are involved in immune system responses, regulating cholesterol efflux and secretion, clearance of LDL from the blood, and activation of the coagulation system. The authors suggest that these 10 proteins can serve as optimal candidates for further research on proteomic biomarkers following radiation exposure [221].

Currently, most studies focus on mice and NHPs as primary research subjects. However, protein expression varies among different species, and the unique characteristics of species in radiation induction must be considered in research. A comparative study of the radiation‐induced proteomic expression profiles in four different species revealed significant changes in insulin‐like growth factor binding protein (IGFBP), cell serine protease, tissue cathepsin, matrix metalloproteinase (MMP), and myosin light chain, which are associated with the symptoms of DNA DSB repair after whole‐body irradiation and damage to the heart and kidneys. The study also identified a previously non‐established radiation exposure biomarker, HIST1H1C (histone H1.2), whose expression was validated through ELISA. Significant dose‐dependent increases in HIST1H1C were found in plasma samples from C57 BL 6 mice collected 24 h after irradiation with doses of 2, 3.5, and 8 Gy, indicating that HIST1H1C may serve as a useful protein biomarker for determining radiation dose within 24 h post‐exposure [222]. Overall, these reports have demonstrated the potential of proteins as reliable biomarkers for high‐dose radiation exposure.

Traditional radiation biology research has focused on DNA damage and repair, mutation induction, and chromosomal rearrangements. However, in addition to interacting with genomic DNA, IR may also alter the structure and function of critical cellular components, activate pro‐inflammatory responses, and ultimately affect cell signaling [223, 224]. Indirect consequences of radiation exposure include the generation of ROS through hydrolysis, enhanced NADPH oxidase activity, and mitochondrial dysfunction leading to cellular lipid damage [67]. Lipid molecules altered after radiation can be identified and quantified on a large scale by metabolic phenotypic analysis, and a radiation‐induced lipidomic metabolic profile can be constructed to screen promising radiation biomarkers [225].

Researchers analyzed lipidomic changes in a mouse radiation model and found that serum levels of ether phosphatidylcholine (PC) significantly increased, while levels of diacyl PCs carrying PUFAs significantly decreased. Upregulated substances primarily derived from the COX and LOX pathways are omega‐6 pro‐inflammatory lipid mediators (e.g., HETEs, PGF 2α, and TBX 2), which play crucial roles in renal and cardiovascular functions and are associated with the development of hypertension. Conversely, downregulated omega‐3 anti‐inflammatory mediators (5‐HEPE and 9‐HoTrE) possess anti‐inflammatory and analgesic properties and can also act as platelet aggregation inhibitors and pulmonary smooth muscle relaxants [226].

Particularly noteworthy was the marked elevation of three polyunsaturated triglycerides (PUFA‐TGs) containing arachidonic acid (20:4, AA) and docosahexaenoic acid (22:6, DHA) acyl chains, suggesting their potential rapid transport to peripheral tissues for storage via enhanced biosynthetic pathways [227]. Further metabolomic analysis revealed elevated concentrations of most LysoGPs post‐irradiation, indicating their potential as sensitive molecular markers for radiation‐induced inflammatory responses [228].Comprehensive lipid profiling showed increased levels of both esterified derivatives and free active forms of AA and DHA, with the former exhibiting more pronounced elevation [227]. From a functional perspective, the enrichment of esterified AA—a key precursor for pro‐inflammatory mediators like prostaglandins—may participate in lipid transport or cellular signal transduction [229, 230]. Conversely, the dynamic changes of DHA, known for its potent anti‐inflammatory properties, may reflect the organism's compensatory regulation against radiation‐induced damage [231].

Of particular significance, the relative decrease in free AA/DHA levels might result from accelerated lipid conversion due to enhanced esterase activity in the inflammatory microenvironment [232]. These findings provide compelling evidence that IR can remodel the global lipid metabolic network to precisely regulate the dynamic balance between inflammatory responses and tissue repair processes. However, the spatiotemporal distribution patterns, functional heterogeneity of radiation‐specific lipid molecules in damage response, and their intricate crosstalk with DNA repair pathways require systematic investigation through integrated multi‐omics analysis and advanced gene‐editing model systems to fully elucidate their biological functions and regulatory networks.

Additionally, acyl‐acyl PC (PC aa C34:1 and PC aa C34:4), lysoPC (lysoPC a C18:0 and lysoPC a C17:0), and sphingomyelins (SM C20:2) in bone marrow cells can serve as markers to distinguish between irradiated groups and control groups. These changes may be associated with oxidative stress responses mediated by Nrf2 induction in osteoblast differentiation‐induced cells, increased IL6 expression, and reduced mitochondrial protein expression in bone marrow stromal cells [233]. Researchers also collected plasma samples from 22 acute myeloid leukemia patients who were hospitalized for bone marrow transplants, both before and after radiation exposure. Acute whole‐body γ‐ray irradiation was performed at doses of 0, 4, 8, and 12 Gy. Ultra‐performance liquid chromatography‐tandem mass spectrometry (UPLC‐MS/MS) and multiple reaction monitoring (MRM) methods were used. A self‐paired study was conducted before and after irradiation, and the metabolic pathways of the potential lipid biomarkers identified mainly included sphingolipid and glycerolipid metabolism, unsaturated fatty acid biosynthesis, and fatty acid degradation and biosynthesis [234].

However, there are still limitations in using animal models to simulate lipid changes following radiation exposure in humans. Due to rapid lipid metabolism changes, lipid indicators typically exhibit excellent sensitivity and specificity within 1 day after lethal dose radiation, but their effectiveness gradually diminishes later. By 7 days, lipid changes in serum can no longer be distinguished from control samples [179]. Therefore, there is still a lot of research space for simulating human serum lipid changes using animal models.

### 3.3 Novel Biosensors and Rapid Detection Technologies

**FIGURE 3:**
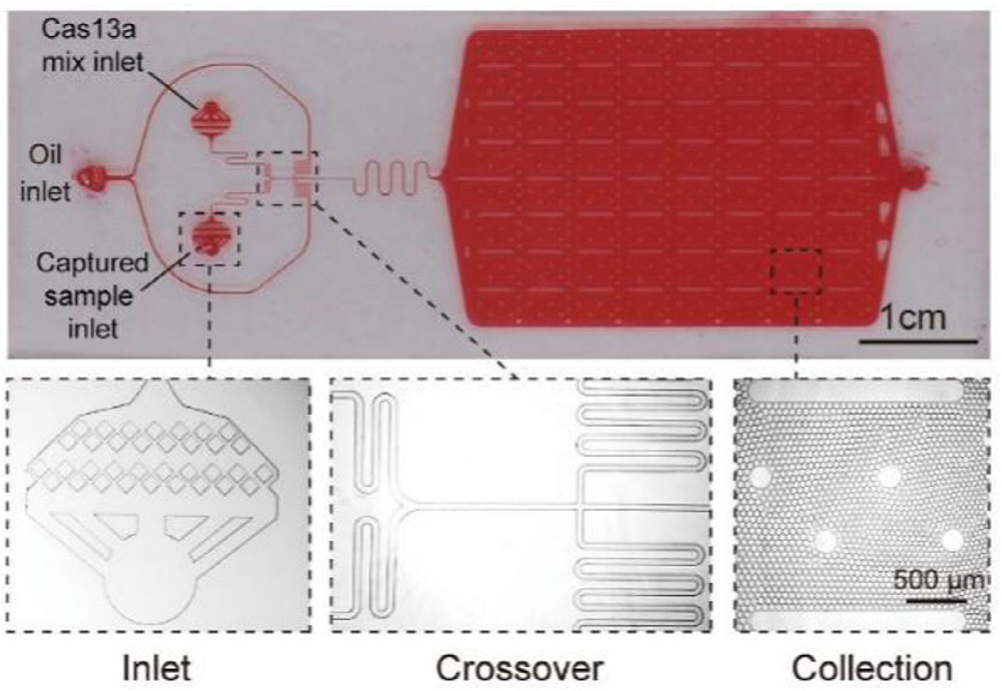
Vertical view of the CM platform and the enlarged images of structural details. Vertical view of the CM platform: The captured sample and Cas13a mix were injected into the chip inlets and mixed in the middle microchannel.

It is reported that the N‐6‐methyladenosine (m6A) content on the nuclear receptor coactivator 4 (NCOA4) gene in PBMCs rapidly increases with IR exposure and that this increase is proportional to the IR dose [172]. To tackle the challenge of achieving high sensitivity and rapid quantification of radiation doses, researchers designed a Cas13a‐microdroplet (CM) platform that can sensitively detect radiation doses within 1 h with an error of less than 0.5 Gy. The platform operates on the principle of generating uniform microdroplets that encapsulate m6A‐containing genes and the CRISPR/Cas13a detection system, wherein Cas13a recognizes the NCOA4 sequence and cleaves the reporter gene, resulting in amplified fluorescent signals within 20 min. The resulting microdroplets effectively reduce the reaction volume from microliters to picoliters, significantly enhancing the likelihood of the reaction. This development has enabled the ultra‐sensitive detection of NCOA4‐m6A at a concentration of 5 copies/µL [235] (Figure 3).

The Biomedical Advanced Research and Development Authority (BARDA), a division of the Assistant Secretary for Preparedness and Response (ASPR) within the U.S. Department of Health and Human Services, has funded biodosimetry projects since 2009 [130]. These projects involve 11 technologies, such as proteomics, gene expression, and DNA damage, aimed at developing high‐throughput qualitative and quantitative biodosimetry methods [236]. However, after evaluating accuracy, feasibility, and cost, only four projects were found to hold further research value.

The Stanford Research Institute (SRI) is developing a lateral flow immunoassay utilizing nitrocellulose strips impregnated with antibodies targeting three proteins: salivary alpha amylase (AMY1A), Fms‐related tyrosine kinase 3 ligand (FLT3L), and monocyte chemotactic protein 1 (MCP1). These proteins are quickly released into the plasma after radiation exposure, persist for at least 14 days, and exhibit a significant dose‐dependent relationship within the range of 0–10 Gy. Research involving both humans and NHPs indicates that SRI has analyzed the performance data from the existing dataset, revealing a specificity of 90% and a sensitivity of 94.3% [237].

### 3.4 Integration of Multi‐Omics Data and Machine Learning Models for Dose Prediction

Predicting radiation doses using radiation‐sensitive genes is the most heavily funded project. DxTerity has created a high‐throughput biodosimetry testing system named REDI‐Dx, which predicts an organism's radiation dose by measuring the expression of a set of radiation response genes. The primary distinction between these two tests is that REDI‐Dx does not amplify targeted RNA through reverse transcription; rather, it converts the RNA into specifically amplifiable DNA segments through the chemical linkage of two consecutively hybridized DNA fragments, followed by amplification via qPCR and size differentiation using capillary electrophoresis. REDI‐Dx has been validated for systematic performance at cutoff values of 2.0 Gy and 6.0 Gy, demonstrating sensitivities and specificities of 98.5% and 90%, respectively, at 2.0 Gy, and 92% and 84%, respectively, at 6.0 Gy. So far, early testing of the devices, algorithms, and operators' use of the assays has shown promise [238].

The abundance of micronuclei (MN) resulting from radiation‐induced DNA breaks in lymphocytes provides a more direct measure of radiation damage [239]. The last high‐throughput quantitative assay funded by HHS is the CytoRADx test developed by ASELL, a highly advanced cytokinesis‐block micronucleus assay (CBMN) that represents an evolved version of earlier CBMN tests [240]. This assay enhances the efficiency of CBMN detection through improved standardized kits, optimized detection protocols, fully automated microscopy and image analysis, and integrated dose prediction. These advancements allow the CytoRADx system to deliver high‐throughput, standardized results without requiring specialized personnel or laboratory‐specific calibration curves [241]. In tests involving human and NHP ex vivo blood samples, the CytoRADx system has shown accurate and precise measurements of radiation exposure ranging from 0 to 8 Gy. However, a limitation of the CBMN assay is that it requires overnight cell culture, leading to longer turnaround times compared to gene expression assays [242].

## 4 Applications in Cancer Therapy

Radiation is a physical agent, which is used to destroy cancer cells. The radiation used is called IR because it forms ions (electrically charged particles) and deposits energy in the cells of the tissues it passes through. This deposited energy can kill cancer cells or cause genetic changes resulting in cancer cell death. Radiation therapy can kill cancer cells by a variety of mechanisms. The main goal of radiation therapy is to deprive cancer cells of their multiplication potential and eventually kill the cancer cells. Cancer cells whose DNA is damaged beyond repair stop dividing and die.

### 4.1 Targeting Radiation‐Induced Cell Death in Therapy

This section comprehensively explores how radiobiological mechanisms translate into therapeutic opportunities and clinical decision‐making. We discuss three key themes: (i) leveraging radiation‐induced cell death to eliminate tumors; (ii) enhancing radiotherapy sensitivity through engineered approaches to broaden the therapeutic window; (iii) achieving personalized treatment using radiation‐responsive biomarkers and radiotherapy diagnostic tools. The core objective is to establish connections between molecular effectors and their microenvironmental context, and between these factors and treatment design, combination therapy strategies, and practice‐relevant endpoints such as tumor control, normal tissue toxicity, and survival rates.

#### 4.1.1 Radiation‐Induced Apoptosis in Tumor Therapy

Apoptosis is a highly regulated process of programmed cell death that is widespread in living organisms and plays an important role in tissue development, immune responses, and pathological states [243]. In radiotherapy, the initiation of apoptosis is closely related to the radiation dose and cell type. Generally low doses of radiation are more likely to induce apoptosis, whereas higher doses may lead to necrosis [244]. As mentioned above, apoptosis is activated mainly by two pathways, endogenous and exogenous, in which the endogenous pathway is initiated mainly by the release of pro‐apoptotic factors from the mitochondria, and the exogenous pathway promotes apoptosis by the activation of caspase family enzymes through ligands binding to the receptor [245]. After tumor cells are exposed to radiation, intracellular Bcl‐2 family members activate pro‐apoptotic factors by modulating mitochondrial membrane permeability and trigger cell death [246].

In radiotherapy, promotion of tumor cell apoptosis by several means can significantly increase the radiation sensitivity of tumor cells. It was shown that the inhibitory effect of far infrared radiation (FIR) from graphene on malignant melanoma (MM) cells was partly achieved by inducing apoptosis. After FIR treatment, the proliferation of B16F10 melanoma cells was inhibited, and the cell cycle was stalled at the G0/G1 phase accompanied by the downregulation of the expression of hypoxia‐associated proteins such as HIF‐1α. Further studies showed that FIR‐induced apoptosis was dependent on the activation of Caspase‐3 and Caspase‐9, which provided a new idea for the application of FIR in tumor therapy [247]. In addition, the natural pigment prodigiosin (PG) can inhibit tumor proliferation and enhance IR sensitivity by promoting apoptosis of colon cancer cells. What's more, PG regulates redox balance, slows tumor growth, and significantly increases the sensitivity of tumor cells to radiation when combined with gamma rays by interfering with the Bcl‐2/caspase‐3 and PPAR‐γ signaling pathways [248]. In recent years, it has also been demonstrated that the balance of the anti‐apoptotic proteins Bcl‐2 and Bcl‐xl and pro‐apoptotic proteins Bax and Bad in the endogenous apoptotic pathway plays an important role in tumor cell survival after radiation [56]. The development of small molecule inhibitors by targeting anti‐apoptotic proteins can enhance the effect of radiotherapy and attenuate the radiation tolerance of tumor cells. In addition, Sirt3, as an important mitochondrial protein, is gradually being focused on its potential in radiosensitization. Studies have shown that the expression level of Sirt3 directly affects the repair ability of mtDNA after IR, and overexpression of Sirt3 increases the radiation resistance of cancer cells, whereas deletion of Sirt3 exacerbates apoptosis, which provides a new target for radiosensitization [249].

#### 4.1.2 Radiation‐Induced Necroptosis in Tumor Therapy

Necroptosis is a programmed mode of necroptotic cell death that exhibits cell membrane rupture and proinflammatory factor release similar to cell necrosis, which can effectively activate the immune system, thus becoming one of the important mechanisms of radiosensitization and radiation protection in tumor therapy [250]. Unlike apoptosis, necroptosis does not rely on caspase activity, but rather is mediated through the cascade activation of key molecules such as RIPK1, RIPK3, and MLKL, which triggers cell membrane rupture and release of cellular contents, thereby activating the immune system [251]. Studies have shown that radiation therapy significantly upregulates the expression of MLKL in tumor cells and activates the RIPK1/RIPK3/MLKL axis through the ZBP1‐mediated pathway, directing tumor cells to undergo necroptosis [252]. This process not only directly causes tumor cell death, but also activates the cGAS‐STING pathway through the release of mtDNA and cytoplasmic DNA, induces type I interferon responses, and enhances CD8⁺ T‐cell responses, which in turn amplifies the antitumor immune effect and further enhances the efficacy of radiotherapy [253]. In addition, it has been demonstrated that knockdown or inhibition of Caspase‐8 may unlock its inhibitory effect on necroptosis and enhance MLKL activation and STING pathway‐mediated immune response, thereby significantly enhancing the efficacy of radiotherapy [254]. In head and neck squamous and colorectal cancer models, necroptosis signaling activated by knockdown of Caspase‐8 significantly increased the sensitivity to radiotherapy [255, 256]. This suggests that increasing radiation‐induced necroptosis is a potential target to block tumor recurrence and enhance the efficacy of radiotherapy. and enhancement of radiotherapy efficacy.

However, it is worth noting that over‐activation of necroptosis may also lead to radiation damage in normal tissues. Therefore, it is also important in radiation protection strategies by regulating necroptosis‐related signals. Nrf2 was found to negatively regulate the RIPK1/RIPK3/MLKL pathway and reduce radiotherapy‐induced damage to healthy rectal tissues [257]. Nrf2 deficiency exacerbated radiorectal damage, whereas its overexpression or the use of a necroptosis inhibitor, Nec‐1, was effective in reducing healthy cell radiosensitivity and reduce radiation‐induced DNA damage and mitochondrial dysfunction [258]. In addition, natural compounds such as saffronin have been shown to alleviate radiological lung injury by inhibiting programmed necrosis‐related genes, such as Tnfrsf10b, which provides a new strategy for radiological normal tissue protection [259].

#### 4.1.3 Radiation‐Induced Pyroptosis in Tumor Therapy

Pyroptosis is characterized by a more pronounced inflammatory response compared with apoptosis and necrosis. Pyroptosis is dependent on the cleavage and activation of Gasdermin protein family members, and in particular, the role of Gasdermin E in tumor cells has received increasing attention [260]. In recent years, an increasing number of studies have revealed that the induction of pyroptosis in radiation therapy may be another potential means of enhancing the sensitivity of tumor radiotherapy [261]. Ionizing radiation can induce the development of pyroptosis in tumor cells by activating the intracellular Caspase‐9/Caspase‐3/GSDME pathway to induce pyroptosis in tumor cells. Studies have shown that IR can effectively initiate cell pyroptosis in tumor cell lines with high expression of GSDME, such as lung cancer, hepatocellular carcinoma, breast cancer, and glioma [262]. In the treatment modality of IR combined with chemotherapeutic agents, such as cisplatin or etoposide, GSDME significantly mediates cell pyroptosis, and this combination therapy enhances the immune response to the tumor by increasing the radiosensitivity of the tumor cells concomitantly with significantly enhanced tumor suppression [263]. The induction of strong immune activation is a typical feature of cellular pyroptosis, which is crucial in radiosensitization. It has been shown that GSDME‐mediated pyroptosis not only kills tumor cells directly through cell death effect, but also indirectly enhances tumor immunosurveillance by promoting the infiltration of CD8+ T cells as well as the release of relevant cytokines [264].

However, in radiation adverse effects such as radiation lung injury, radiation‐induced cell pyroptosis may also exacerbate the inflammatory response and fibrosis [265]. Ionizing radiation can trigger the release of double‐stranded DNA in the lumen of the bronchoalveolar lumen, activating the cGAS‐STING pathway, which in turn triggers the activation of the NLRP3 inflammatory vesicle, provoking pyroptosis and exacerbating the exacerbation of inflammation and fibrosis. It has been found that inhibiting the activity of cGAS or STING, or applying drugs such as recombinant human DNase I Pulmozyme, can effectively attenuate radiological lung injury and alleviate radiation‐induced lung damage [266]. Therefore, how to enhance the effectiveness of tumor treatment while reducing radiation damage to normal tissues has become a challenge in radiation therapy.

#### 4.1.4 Radiation‐Induced Ferroptosis in Tumor Therapy

Ferroptosis is an iron‐dependent mode of cell death characterized by the accumulation of intracellular iron with lipid peroxides, which ultimately leads to cell death [267]. As a novel type of programmed cell death, ferroptosis is increasingly being studied in cancer therapy. Studies have shown that IR can activate ferroptosis‐related pathways while inducing tumor cell death, which provides new ideas to improve the therapeutic efficacy of radiotherapy [268].

Ionizing radiation induces excessive accumulation of intracellular iron and lipid peroxidation, thereby triggering ferroptosis. Studies have shown that the intracellular levels of lipid peroxides are significantly elevated after IR, and the occurrence of ferroptosis has been observed especially in a variety of cancer cells such as carcinoma of the lung, breast, esophagus, ovary, kidney, fibrosarcoma, and others [269]. These tumor cells exhibit increased expression of the lipid peroxidation markers malondialdehyde and 4‐HNE, as well as of the ferroptosis markers ACSL4 and PTGS2. In terms of radiosensitization, ferroptosis inducers can be used as potential radiosensitizers. Krishan et al. [270] found that ferroptosis inducers that inhibit the amino acid transporter protein SLC7A11 had a significant radiosensitizing effect on tumors with p53 mutations or deletions. Feng et al. [271] found that Nrf2 inhibited ferroptosis by promoting the overexpression of SLC7A11, thereby increasing the radiosensitivity of esophageal squamous cell carcinoma. Therefore, blocking the Nrf2/SLC7A11/ferroptosis pathway may increase the radiosensitivity of cancer cells. In addition, due to the symbiotic relationship between ferroptosis and autophagy pathway, IR promotes ferroptosis by inducing mitochondrial autophagy and increasing intracellular free fatty acids and lipid peroxidation. The activation of ferroptosis by IR is more pronounced in cells with higher levels of autophagy [64].

The induction of ferroptosis not only enhances the sensitivity of tumor cells to radiotherapy, but may also provide a new therapeutic strategy for anti‐radiotherapy resistance. For example, RRFERV long‐chain ncRNA activates the Hippo signaling pathway by stabilizing TEAD1, while inducing transcriptional enhancement of ferroptosis by ACSL4. This sensitized tumor cells to ferroptosis inducers and reversed radiotherapy resistance [272]. Similarly, SOCS2 induced ferroptosis in hepatocellular carcinoma cells by promoting K48 polyubiquitination degradation of SLC7A11 [273]. Furthermore, FSP1‐mediated ferroptosis in lung carcinoma cells and ameliorated radiotherapy resistance through activation of the CoQ‐FSP1 axis [274].

In terms of side effect control of radiotherapy, IR may likewise trigger the occurrence of ferroptosis in normal cells, leading to radiation damage. Elevated lipid peroxidation markers such as 4‐HNE and MDA were observed in healthy intestinal tissues after IR, suggesting activation of ferroptosis [275]. Ferroptosis leads to radiation injury in healthy intestinal tissues through the STAT1‐IRF1‐ACSL4 pathway. The use of Fer‐1, an inhibitor of ferroptosis, significantly attenuates radioactive intestinal damage and improves intestinal function in murine models, providing a potential strategy for combating radiation‐induced normal tissue damage [276].

### 4.2 Radiosensitization Strategies

#### 4.2.1 STING Agonists and Radiosensitization

RT mainly exerts its therapeutic effect through DNA damage induced by IR [277]. In addition, RT can also induce immunogenic cell death (ICD) and activate the cGAS–STING pathway to activate the systemic antitumor immune response [278], further increasing its efficacy.  Interestingly, RT has been observed to induce the “abscopal effect” in various cancer types, including breast cancer [279], melanoma [280],  lymphoma [281], renal cell carcinoma [282], and other metastatic solid tumors [283, 284]. However, due to the immunosuppressive TME and problems such as tumor immune escape by immune checkpoint blockade (ICB) and adoptive cell transfer (ACT) [285], the activation of the cGAS–STING pathway by RT remains transient and suboptimal, and it is unable to maintain strong antitumor immunity. Therefore, combining RT with STING agonists may benefit traditional therapies by amplifying tumor immunogenicity and counteracting immune evasion. The latest research shows that enhancing the activation of the cGAS–STING pathway can enhance the efficacy of RT while alleviating immune suppression within the TME [286]. Currently, STING agonists are usually used for high‐dose intratumoral injections to treat tumors. Due to poor cell membrane permeability, poor targeting, poor stability, and low bioavailability, they have inevitable side effects [287].

Nanomedicine provides a promising approach for the combination of RT and STING agonists [288]. On the one hand, nanoscale radiosensitizers with high atomic number (High‐Z) metals such as gold (Au) and hafnium (Hf) can induce DNA damage by depositing radiation energy and generating ROS, thereby improving the efficacy of RT. Notably, functionalized hafnium oxide (HfO_2_) nanoparticles (NBTXR3) showed significant clinical benefits for patients with locally advanced soft tissue sarcoma in a European phase II/III clinical trial (NCT02379845) [289]. On the other hand, nanocarriers can selectively deliver STING agonists to enhance radioimmunotherapy while minimizing off‐target toxicity. The combination of RT and STING agonists exhibits dual effects of radiosensitization and immunostimulation, induces long‐lasting immune memory, and reshapes the TME.

#### 4.2.2 Nuclear Receptor Modulators and Radiosensitization

Increasing evidence suggests that nuclear receptor modulators (including agonists and antagonists) play a key role as promising radiosensitizers in radiotherapy through various mechanisms. Abnormal expression of nuclear receptors may be one of the key factors in tumorigenesis and the development of tumor radiation resistance [290, 291]. Long and Campbell analyzed the breast tissue data of 1905 patients with in situ breast cancer and 113 healthy subjects in The Cancer Genome Atlas (TCGA) database and reported that 42 nuclear receptors were abnormally expressed in tumors compared with normal tissues [292]. To date, many nuclear receptor‐based drugs (including Estrogen receptor, Androgen receptor, Liver X receptor, Retinoid X receptor, Peroxisome proliferator‐activated receptor, and glucocorticoid receptor) have entered different stages of clinical trials in cancer patients, as summarized by Yang Z et al. [293]. These drugs include the selective ER modulator bazedoxifene for patients with ductal carcinoma (NCT02694809), the selective AR degrader ARV‐110 for castration‐resistant prostate cancer (CRPC) (NCT03888612), the LXR agonist RGX‐104 for patients with advanced solid malignancies and lymphoma (NCT02922764), the RXR agonist bexarotene for recurrent or refractory cutaneous T‐cell lymphoma (NCT01134341), the PPAR agonist pioglitazone for pancreatic cancer (NCT01838317), and the GR antagonist relacorilant in combination with albumin‐bound paclitaxel for patients with solid tumors (NCT02762981) [293].

In addition, clinical studies on the treatment of patients with these drugs in combination with radiotherapy have been reported. For example, tamoxifen combined with radiotherapy is used to reduce local recurrence after breast‐conserving surgery, dexamethasone and whole‐brain radiotherapy are used to treat NSCLC brain metastases, and enzalutamide combined with radiotherapy is used to treat intermediate‐ and high‐risk prostate cancer [294, 295, 296, 297]. It is expected that novel nuclear receptor agonists and inhibitors will be used in the future to sensitize clinical tumors to radiotherapy.

#### 4.2.3 FLASH and Radiosensitization

In recent years, FLASH radiotherapy (FLASH‐RT), which uses a linear electron accelerator to achieve ultra‐high dose rate irradiation with an average dose rate exceeding 40 Gy/s within a delivery time of less than 200 ms, has attracted increasing attention. FLASH‐RT is characterized by instantaneous, ultra‐high dose, and single‐shot irradiation, which can effectively shorten the radiotherapy treatment time, improve the tolerance of normal tissues, and has a high therapeutic effect [298, 299]. In 2019, FLASH‐RT was used for the first time in the clinical treatment of a patient with multi‐drug resistant T‐cell cutaneous lymphoma [300]. In 2023, a non‐randomized clinical study on proton FLASH‐RT for bone metastases was conducted, supporting the application of FLASH‐RT in clinical treatment [301]. Studies have shown that FLASH‐RT induces less ROS and promotes the preservation of mitochondrial integrity and function, which helps to attenuate the apoptotic pathway in normal tissues and reduce damage [302]. However, the molecular radiobiology behind the FLASH effect has not been fully elucidated, and further experiments are needed to understand the biological response.

#### 4.2.4 Radiation‐Induced Senescence in Tumor Therapy

Cellular senescence is the process by which cells undergo permanent proliferative arrest, accompanied by a series of phenotypic changes, including flattening of cellular morphology, vacuolization and changes in cytoplasmic granularity, and abnormalities in organelles. Senescent cells functionally exhibit the release of SASPs with autocrine, paracrine, and endocrine activities, and these factors not only affect the senescent cells themselves, but may also affect the surrounding healthy cells [303]. In tumor therapy, cellular senescence is not only a common nonlethal cellular endpoint after IR induction, but also its dual roles in radiotherapy have triggered an increasing interest. The induction or inhibition of cellular senescence may have an important impact on the efficacy of tumor therapy as well as the protection of normal tissues.

During radiotherapy, IR activates the p53 gene through DNA damage, which in turn upregulates p21 expression and inhibits the activity of the CDK–cyclin complex, leading to cell cycle arrest. This process forms a block at multiple cell cycle points in the G1 and S phases, as well as in the G2 and M phases, thereby contributing to the entry of cells into the senescence state [304]. With the accumulation of senescent cells, radiation‐induced oxidative stress and activation of PKC are also able to increase the expression of p16, which further enhances the process of cellular senescence. This senescence process has an inhibitory effect on tumor cell proliferation at a certain level, but with the passage of time, senescent cells may adversely affect tumor recurrence and metastasis. Especially in the later stages of radiotherapy, the pro‐inflammatory factors secreted by senescent cells may create an immune microenvironment that supports tumor recurrence [33].

Targeting the removal or inhibition of senescent cells has emerged as a potential radiosensitization strategy. Senolytics that selectively remove senescent cells and senostatics that inhibit the activity of senescent cells are the two main directions of current research. Senolytics include a number of small molecules, peptides, and antibodies, such as dasatinib, quercetin, and navitoclax [305]. These drugs can reduce normal tissue damage and improve tumor outcome after radiotherapy by selectively removing senescent cells. For example, Navitoclax has shown significant effects in reducing radiation‐induced pulmonary fibrosis and hematotoxicity, as well as being able to delay recurrence of malignant gliomas by eliminating IR‐induced senescence of astrocytes [39]. Unlike senolytics, the mechanism of senostatics is to slow down the spread of senescent cells by inhibiting their paracrine signaling. By modulating signals such as the mTOR pathway and mitochondrial function, senostatics can indirectly influence the effects of senescence cells. For example, drugs such as rapamycin and metformin have been shown to inhibit the spread of senescent cells [306]. These drugs not only help protect normal tissues from IR damage but may also increase the sensitivity of tumor cells to radiotherapy. The rational use of drugs such as senolytics and senostatics, by removing or inhibiting senescent cells, not only helps to enhance the efficacy of radiotherapy but also provides a new therapeutic strategy for the protection of normal tissues. The radiosensitizing agents used clinically are detailed in Table 4.

**TABLE 4:** Some of investigational and clinically applied radiosensitizing agents.

Name	Category	Types of cancer	Mechanisms	Phase	Refs.
CS‐Mn	Microparticles	Diverse cancer entities	Promotes tumor infiltration and activation of innate immunity	Preclinical study	[342]
Salidroside	Small molecule	Diverse cancer entities	Arrests cells in the radiosensitive G2/M phase, amplifies reactive oxygen species (ROS) generation, and exacerbates DNA damage	Preclinical study	[343]
BSCgal	Nanoparticle	HNSCC mouse model	Promotes antitumor leukocytes, upregulates cytotoxic granzyme B, and reduces immunosuppressive cell populations	Preclinical study	[344]
CBL@HfO_2_	Nanoparticle	Diverse cancer entities	Promotes Z‐DNA cleavage	Preclinical study	[345]
PWCu nanocapsules	Nanoparticle	Human cervical cancer tissues	Upregulates key regulators of cuproptosis	Preclinical study	[346]
HA@AuNC@CO	Nanoparticle	Diverse cancer entities	Generates ROS to inactivate cancer cells and inhibits the glycolytic pathway	Preclinical study	[347]
APR‐246	Small molecule	HCT116	Restores p53 function, modulates the cell cycle, and induces apoptosis	Preclinical study	[348]
Atovaquone	Small molecule	DMG mouse model	Targets mitochondrial complex III to inhibit mitochondrial metabolism in cancer cells	Preclinical study	[349]
Enzalutamide	Small molecule	Intermediate‐risk prostate cancer	Inhibit the proliferation of prostate cancer cells and induce their apoptosis	Clinical application	[331]
Ta@PVP NPs	Nanoparticle	Chordoma	Generates ROS and induces oxidative stress	Preclinical study	[350]
MitoQ	Small molecule	Orthotopic breast cancer mouse model	Reduces mitochondrial oxygen consumption in cancer cells	Phase I clinical trial	[351]
AZD1390	Small molecule	Glioblastoma	Inhibits ATM, modulates the cell cycle, and promotes cancer cell apoptosis	Phase I clinical trial	[352]
Quinoline‐indole‐Schiff base derivative 10E	Small molecule	NSCLC	Inhibit cancer cell proliferation and DNA damage repair	Preclinical study	[353]
Napabucasin	Small molecule	DMG	Generate and target NQO1	Phase III clinical trial	[354]
sMnAu NAs	Nanoparticle	Diverse cancer entities	Activate STING pathway and potentiate the efficacy of immunotherapy	Preclinical study	[355]
Peposertib	Small molecule	MBM	Downregulate DNA‐PKcs and inhibit the DNA repair pathway	Preclinical study	[356]
Olaparib	Small molecule	Breast cancer	Inhibit PARP enzyme	Clinical application	[357]
Bevacizumab	Monoclonal antibody	Diverse cancer entities	Modulates vascular permeability, angiogenesis, and endothelial cell migration and survival	Clinical application	[358]
Cetuximab	Monoclonal antibody	HNSCC	Inhibits tumor proliferation, induces apoptosis, disrupts DNA damage repair, and modulates the cell cycle	Clinical application	[359]
Cisplatin	Small molecule	Diverse cancer entities	Induces DNA damage, inhibits replication and transcription, blocks DDR pathway, and modulates the cell cycle	Clinical application	[360]
Ibrutinib	Small molecule	Diverse cancer entities	Regulate the cell cycle and induce apoptosis	Clinical application	[361]
Curcumin	Small molecule	Diverse cancer entities	Inhibit tumor cell proliferation, induce apoptosis, block invasion, metastasis and angiogenesis, and regulate the tumor microenvironment	Preclinical study	[362]
Alvespimycin	Small molecule	Diverse cancer entities	Inhibit tumor cell proliferation and promote apoptosis	Preclinical study	[363]
Patritumab deruxtecan	Hybrid pharmaceutical	Diverse cancer entities	Target HER3 and induce apoptosis	Clinical application	[364]
Paclitaxel	Small molecule	Diverse cancer entities	Stabilize microtubules, inhibit mitosis, and regulate the immune microenvironment	Clinical application	[365]
Nimotuzumab	Monoclonal antibody	HNSCC NPC	Target EGFR and inhibit tumor growth	Clinical application	[366]

Abbreviations: DMG: diffuse midline glioma; HCT116: human colorectal carcinoma cell line; HER3: human epidermal growth factor receptor 3; HNSCC: head and neck squamous cell carcinoma; MBM: melanoma brain metastases; NPC: nasopharyngeal carcinomathe; NSCLC: non‐small cell lung cancer.

### 4.3 Radiation‐Based Theranostics: From Diagnosis to Treatment Guidance

In the development of radiosensitization strategies, based on the multi‐omics characteristics of the TME (high expression of LDHA, activity of HIF‐1α, extracellular acidification), subpopulations resistant to radiotherapy can be identified, guiding the combined use of targeted drugs such as MCT4 inhibitors and tirapazamine. By inhibiting lactate efflux or enhancing the generation of free radicals in the hypoxic area, the radiobiological effect can be improved (the biologically effective dose [BED] is increased by 1.5–2 times) [307].

In the field of radionuclide‐targeted therapy, ^177^Lu‐PSMA‐617 can precisely kill prostate cancer cells (PSMA⁺) through β^−^ particles. The VISION trial shows that the median OS is prolonged by 4 months [308]. Similar mechanisms have been extended to the treatment of lymphoma with ⁹⁰Y‐CD20 and the treatment of neuroendocrine tumors with ^177^Lu‐DOTATATE [309, 310]. Metabolomics CRISPR screening has revealed the regulatory mechanism of the lipoylation‐TCA cycle axis: LIPT1‐mediated lipoylation of mitochondrial 2‐keto acid dehydrogenase maintains the function of the TCA cycle. Its inhibition (using CPI‐613) leads to the accumulation of 2‐hydroxyglutaric acid (2‐HG). Through the epigenetic modification of H3K9me3, the recruitment of the TIP60‐ATM complex is blocked, and HR repair is inhibited (the HR defect rate is increased by 68%), significantly enhancing the radiosensitivity of non‐small cell lung cancer (the survival fraction is reduced to 0.23 ± 0.05) [311]. Research on colorectal cancer has revealed the dual regulatory role of the chromatin remodeling factor ATRX: The deletion of ATRX upregulates the function of p53 through the Daxx/MDM2 pathway (the expression of p21 is increased by 3.2 times), and at the same time inhibits the activation of the ATM/Chk2 pathway (the number of γ‐H2AX foci is reduced by 42%). In p53‐deficient HCT116 cells, it synergistically enhances radiosensitivity (the surviving fraction at 2 Gy (SF2) is reduced from 0.56 to 0.31) [312]. This effect is accompanied by an increase in the apoptosis rate (the number of Annexin V⁺ cells is increased by 2.8 times) and a decrease in senescence (the number of SA‐β‐gal⁺ cells is reduced by 57%).

At the level of translational medicine, the combination of the ctDNA methylation profile (with a detection limit of 0.01% minimal residual disease [MRD]) and the FDG‐PET metabolic volume parameters (for those with a metabolic tumor volume [MTV] ≤ 10 cm^3^, the 2‐year progression‐free survival [PFS] is 85% compared with 34%) has constructed a full‐process system from treatment decision‐making to prognosis assessment [313, 314]. However, it is necessary to address the translational bottlenecks such as individual epigenetic heterogeneity (e.g. the methylation status of BRCA1 affects the response to PARP inhibitors) and the long‐term toxicity of radionuclide therapy.

The application of radiation biomarkers in the field of tumor diagnosis and treatment is driving the innovation of the precision oncology paradigm.

In early diagnosis, quantitative analysis of DNA damage biomarkers such as gamma H2AX and 8‐oxoguanine can provide potential molecular evidence for early cancer screening [315, 316, 317]. Meanwhile, the metabolic imaging agent ^18^F‐FDG, through PET‐CT technology, can visualize the abnormal glycolytic activity (Warburg effect) of tumor cells, enabling the spatial localization of tumors and the grading of their malignancy [318]. In the efficacy monitoring stage, liquid biopsy technology can track the dynamic changes of tumor related mutation profiles (such as TP53, KRAS) in circulating tumor DNA (ctDNA) to monitor tumor burden in real time and evaluate treatment response [319, 320]; The activation level of caspase‐3 can specifically reflect the degree of initiation of tumor cell apoptosis, and combined with DNA damage repair marker analysis, it can provide reference for predicting radiotherapy sensitivity [321]. It is particularly noteworthy that the spatial heterogeneous distribution of the TME biomarker HIF‐1α can precisely locate the hypoxic niches resistant to radiotherapy through multimodal imaging (such as ⁶⁴Cu‐ATSM PET) [322], and then guide the implementation of the radiomics‐driven Dose Painting technology, achieving personalized radiotherapy throughout the whole process from the delineation of the biological target volume to the optimization of the dose gradient [102, 323].

The above biomarkers integrate multidimensional information such as molecular damage response (DNA repair defects), metabolic reprogramming (abnormalities in glucose/lipid metabolism), and microenvironment remodeling (hypoxia/acidification/immunosuppression), constructing a radiation response biological signature spectrum and providing cross‐scale decision‐making basis for precision tumor diagnosis and treatment.

## 5 Discussion and Future Perspectives

Over the past few decades, with advances in biological sciences and continuous innovations in detection technologies, humanity's understanding of radiation has gradually evolved from limited knowledge to greater clarity. People no longer simply fear the hazards of radiation but instead explore its potential beneficial effects. However, despite this progress, our current comprehension of radiation remains basic. Numerous critical scientific questions remain unclear and await researchers who can unravel them one by one. In the important field of tumor radiotherapy, the mechanisms underlying tumor radioresistance are still complex, with many aspects yet to be fully elucidated [324]. Meanwhile, existing radiotherapy approaches still require improvement to achieve more precise control over radiation damage to normal tissues. In recent years, FLASH‐RT has emerged. Studies show that it can effectively kill tumor cells while significantly reducing radiation damage to normal tissues, making it a promising focal point and breakthrough area in future radiotherapy research [325, 326].

**FIGURE 4:**
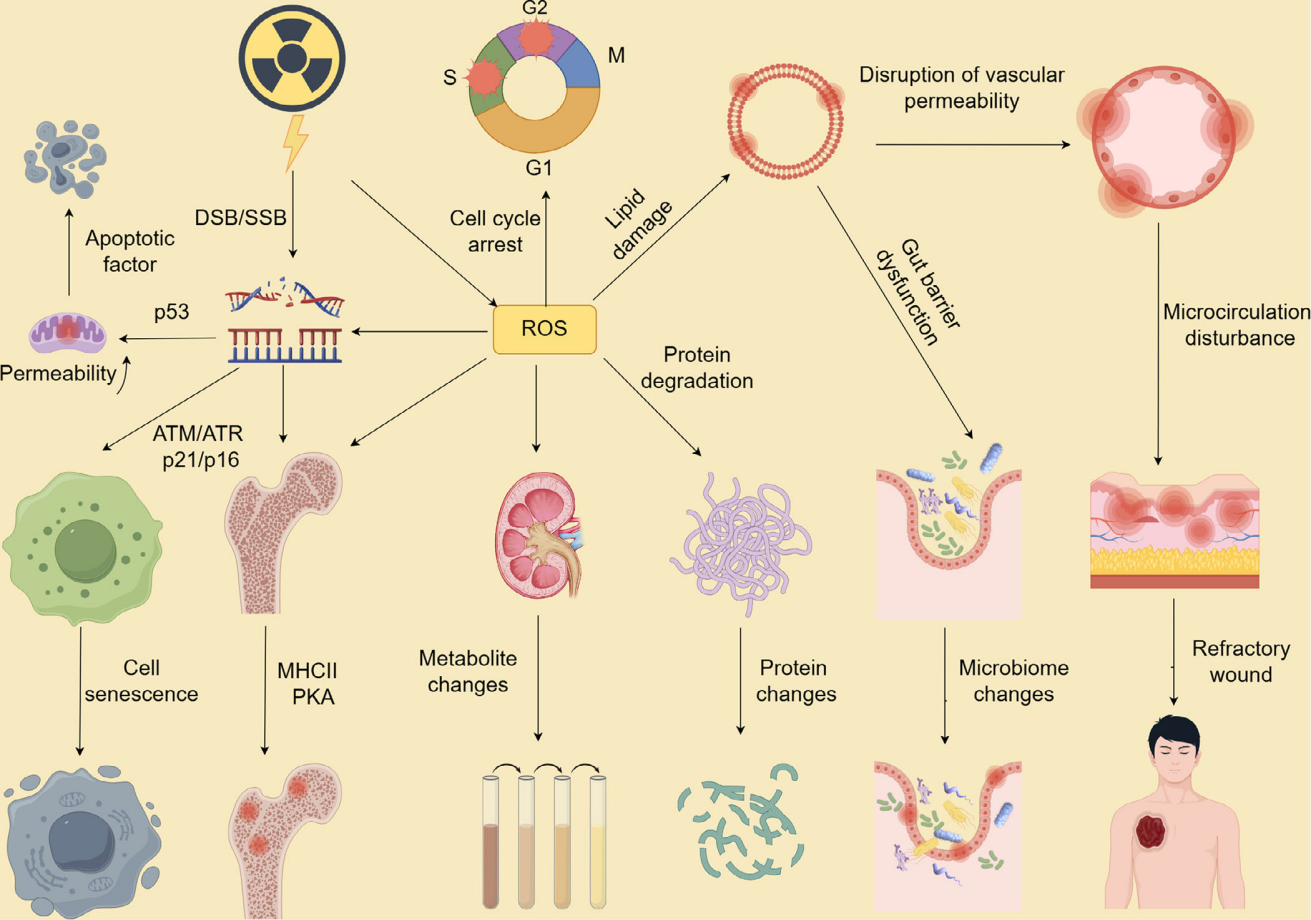
Overview of the main effects of radiation exposure on human health. Schematic diagram of radiation damage to tissues, organs, cells, and molecular effects.

Additionally, the repair of radiation‐induced damage to normal tissues remains a major challenge in the medical field [327]. Currently, the specific repair mechanisms involved in radiation damage are not yet fully understood. This necessitates further research into the molecular and cellular bases of radiation damage repair to provide more solid theoretical support for clinical treatment (Figure 4). Radiation biodosimeters are invaluable in predicting radiation doses in populations affected by large‐scale nuclear accidents. When a nuclear accident occurs, accurately assessing the radiation doses received by the affected population is critical to formulating scientific and rational medical treatment plans, judging prognosis accurately, and optimizing medical resource allocation. Traditional biodosimetry methods, such as dicentric chromosome analysis and lymphocyte micronucleus assays, have a long history of practical use but reveal significant limitations when faced with the high‐throughput screening demands of large affected populations [130, 328].

With the rapid advancement of technology, novel radiation biodosimetry methods continue to emerge. These include detection technologies based on changes in radiation‐sensitive gene expression and precise measurement techniques for specific protein and small‐molecule biomarker levels. However, current research remains at the laboratory stage, and no radiation biodosimetry technology is yet available for large‐scale population screening [329]. As research progresses, these innovative methods are expected to enable rapid and accurate assessment of radiation doses in large populations affected by radiation. This will provide robust technical support for nuclear accident emergency rescue operations. In the future, precision and personalized treatments will undoubtedly become core trends in radiation applications.

As imaging technologies and dose calculation models become increasingly advanced and precise, future radiotherapy is expected to achieve highly precise targeting of tumor volumes and accurate dose delivery. This will thereby minimize collateral damage to surrounding normal tissues [330, 331]. Tailoring radiotherapy plans based on multidimensional information, such as patients' genetic characteristics, tumor biological characteristics, and overall physical condition—including performance status and comorbidities—will become a key pathway to enhancing treatment efficacy and reducing adverse effects [332, 333]. The realization of the ambitious goal of developing optimal treatment strategies for each patient urgently requires deep interdisciplinary integration across radiation oncology, molecular biology, imaging, bioinformatics, and other fields, as well as the combination of data from multiple sources [334]. To advance the implementation of this vision, strengthening basic and clinical translational research in radiotherapy has become imperative. Basic research should focus on an in‐depth analysis of the fine details of radiation‐induced biological effects at the molecular and cellular levels, identifying more potential therapeutic targets and biomarkers to establish a solid theoretical foundation for precision and personalized treatment. Clinical translational research needs to bridge the gap between basic research and clinical practice. It should accelerate the translation of novel radiotherapy technologies, biodosimeters, and innovative treatment plans from the laboratory to clinical application, while actively conducting large‐scale, high‐quality clinical trials to rigorously verify their safety and efficacy in meeting the evolving needs of clinical practice.

## Author Contributions

C.S., L.M., and Z.H. conceived and designed the project. Z.H. and L.M. drafted the manuscript. L.M., Z.H., and Y.C. analyzed the data and drafted the manuscript. C.S. and L.M. edited the manuscript. Y.S. and D.S. participated in the revision of the manuscript. All authors have read and approved the final manuscript.

## Conflicts of Interest

The authors declare no conflicts of interest.

## Data Availability

All data generated and/or analyzed during the current study are included in this published article.
